# Transcriptional regulation of bark freezing tolerance in apple (*Malus domestica* Borkh.)

**DOI:** 10.1038/s41438-020-00432-8

**Published:** 2020-12-01

**Authors:** Yinghai Liang, Shanshan Wang, Chenhui Zhao, Xinwei Ma, Yiyong Zhao, Jing Shao, Yuebo Li, Honglian Li, Hongwei Song, Hong Ma, Hao Li, Bingbing Zhang, Liangsheng Zhang

**Affiliations:** 1 Institute of Pomology, Jilin Academy of Agricultural Sciences, 136100 Gongzhuling, People’s Republic of China; 2 Department of Biology, Eberly College of Science, and The Huck Institutes of the Life Sciences, The Pennsylvania State University, University Park, PA 16802 USA; 3 Ministry of Education Key Laboratory of Biodiversity Sciences and Ecological Engineering, Collaborative Innovation Center for Genetics and Development, Institute of Biodiversity Sciences, Institute of Plant Biology, Center for Evolutionary Biology, School of Life Sciences, Fudan University, 200438 Shanghai, People’s Republic of China; 4 Genomics and Genetic Engineering Laboratory of Ornamental Plants, College of Agriculture and Biotechnology, Zhejiang University, 310058 Hangzhou, People’s Republic of China

**Keywords:** Transcriptomics, Abiotic

## Abstract

Freezing tolerance is a significant trait in plants that grow in cold environments and survive through the winter. Apple (*Malus domestica* Borkh.) is a cold-tolerant fruit tree, and the cold tolerance of its bark is important for its survival at low temperatures. However, little is known about the gene activity related to its freezing tolerance. To better understand the gene expression and regulation properties of freezing tolerance in dormant apple trees, we analyzed the transcriptomic divergences in the bark from 1-year-old branches of two apple cultivars, “Golden Delicious” (G) and “Jinhong” (H), which have different levels of cold resistance, under chilling and freezing treatments. “H” can safely overwinter below −30 °C in extremely low-temperature regions, whereas “G” experiences severe freezing damage and death in similar environments. Based on 28 bark transcriptomes (from the epidermis, phloem, and cambium) from 1-year-old branches under seven temperature treatments (from 4 to −29 °C), we identified 4173 and 7734 differentially expressed genes (DEGs) in “G” and “H”, respectively, between the chilling and freezing treatments. A gene coexpression network was constructed according to this expression information using weighted gene correlation network analysis (WGCNA), and seven biologically meaningful coexpression modules were identified from the network. The expression profiles of the genes from these modules suggested the gene regulatory pathways that are responsible for the chilling and freezing stress responses of “G” and/or “H.” Module 7 was probably related to freezing acclimation and freezing damage in “H” at the lower temperatures. This module contained more interconnected hub transcription factors (TFs) and cold-responsive genes (CORs). Modules 6 and 7 contained C-repeat binding factor (CBF) TFs, and many CBF-dependent homologs were identified as hub genes. We also found that some hub TFs had higher intramodular connectivity (*K*_ME_) and gene significance (GS) than *CBFs*. Specifically, most hub TFs in modules 6 and 7 were activated at the beginning of the early freezing stress phase and maintained upregulated expression during the whole freezing stress period in “G” and “H”. The upregulation of DEGs related to methionine and carbohydrate biosynthetic processes in “H” under more severe freezing stress supported the maintenance of homeostasis in the cellular membrane. This study improves our understanding of the transcriptional regulation patterns underlying freezing tolerance in the bark of apple branches.

## Introduction

The domesticated apple (*Malus domestica* Borkh.: Rosaceae) is one of the most abundant fruit crops in the world^1,2^. Apple trees are also cold-tolerant and can be cultivated in cold regions. The geographical distribution and sustainable production of apple crops, however, is still limited by freezing injury events caused by low temperatures^3,4^. Therefore, an understanding of the genetics and mechanisms of cold tolerance may ultimately lead to improved cold resistance and productivity through breeding or genetic engineering^5,6^.

Cold tolerance represents, in a general sense, the ability of plants to withstand and adapt to chilling (0–18 °C) and freezing (<0 °C) temperatures^7,8^. To overcome freezing stress, many temperate plant species have evolved a sophisticated process, called cold acclimation, by which plants can acquire freezing tolerance after being exposed to low and nonfreezing temperatures^9,10^. This process involves many physiological and biochemical changes and allows hardy plants to activate expression of many transcription factors (TFs) and cold-responsive genes (CORs) to resist freezing stress^9–11^. In the model plant *Arabidopsis thaliana* (hereafter Arabidopsis), the C-repeat binding factor (CBF) regulatory pathway, which plays a vital role in improving cold tolerance by regulating hundreds of CORs, has been extensively studied in the last two decades^7,8,12,13^. *CBF* genes encode APETALA2/ethylene-responsive factor (AP2/ERF)-type TFs, which are able to specifically bind to the C-repeat (CRT)/dehydration-responsive element (DRE; G/ACCGAC)^14^. The expression of downstream COR genes was rapidly induced by *CBF* genes when Arabidopsis plants were exposed to low temperatures (4 °C)^15^. More than two-thirds of COR genes are coregulated by two or three *CBFs*, although each *CBF* regulates different sets of downstream genes^16^. Zhao et al.^13^ highlighted that *CBF* genes activated 414 COR genes through genetic analyses and provided evidence that CBF-independent pathways contributed to cold acclimation^13^. Moreover, CBF-independent regulatory pathways contributed substantially to cold tolerance in Arabidopsis^13,17,18^. For example, five CBF-independent TFs (*HSFC1*, *ZAT12*, *ZAT10*, *CZF1*, and *ZF*) could regulate COR genes when the five factors were overexpressed in transgenic plants^17^.

Compared to Arabidopsis, which can only acclimate to temperatures below 0 °C for a short time^19,20^, many undomesticated woody plants that are native to cold northern regions are able to survive for several months at subzero temperatures^21^ and withstand temperatures as low as −40 °C^21,22^. Therefore, it is relevant to explore the common and specific gene regulation networks related to cold tolerance in woody plants during dormancy^6,23^. At present, functional genomic studies on cold tolerance are mainly focused on fruit crops, such as apple^24^, blueberry, peach, and grape, in the temperate zone, and use EST sequencing, complementary DNA (cDNA) microarrays, and proteomic two-dimensional methods^5^. In apple, five *CBF* genes (*MdCBF1*–*5*) have been identified^6,25^, and some CBF-related TFs, such as *MdCIbHLH1*^3^, *MdNAC029*/*MdNAP*^3^, and *MdMYB23*^26^, have been shown to improve cold tolerance in transgenic plants. However, it remains unclear whether the CBF pathway is involved in freezing damage in dormant apple branches at temperatures below −4 °C.

Weighted gene correlation network analysis (WGCNA) is a useful procedure for classifying genes via the hierarchical clustering of genes in a coexpression network^27^. This procedure has been widely used in various biological studies^28–30^. In a coexpression network, the nodes represent genes, and the edges (also known as the degree or connectivity) between genes represent the total strength of their connections with the other genes in the network^27^. Clusters of highly interconnected nodes identified from a network are called coexpression modules and may reveal actual interactive groups of genes, such as gene regulation pathways^27^, at the system scale. Moreover, principal component analysis (PCA) has been incorporated into the WGCNA algorithm for the performance of coexpression network studies. PCA is a powerful approach for exploring the characteristics of high-dimensional data, such as transcriptome data^31,32^. It is a mathematical procedure that uses an orthogonal conversion to transform thousands of (possibly) correlated variables into a (smaller) set of orthogonal, uncorrelated axes called principal components. A gene coexpression module eigengene can be expressed as the first principal component of the samples^31,32^, and the eigenvectors from the original transcriptome data show the greatest variation along the axis direction of that principal component. The eigengene can represent the gene expression profiles in a module and reveal the module’s biological meaning^27,33^. The intramodular connectivity (*K*_ME_) is the module eigengene-based network connectivity (see the “Materials and Methods” section) and reveals how connected a gene is with other module genes^27^. Hub genes inside coexpression modules tend to have high intramodular connectivity (*K*_ME_). Specifically, a gene in a given module can also be correlated to sample traits using eigengene network methodology, and the correlated coefficient is defined as the gene significance (GS), which represents the gene’s biological importance^27^. Intramodular hub genes tend to have high GS^27^. The higher the absolute value of GS is, the more biologically important this gene is^27,33,34^.

**Fig. 1:**
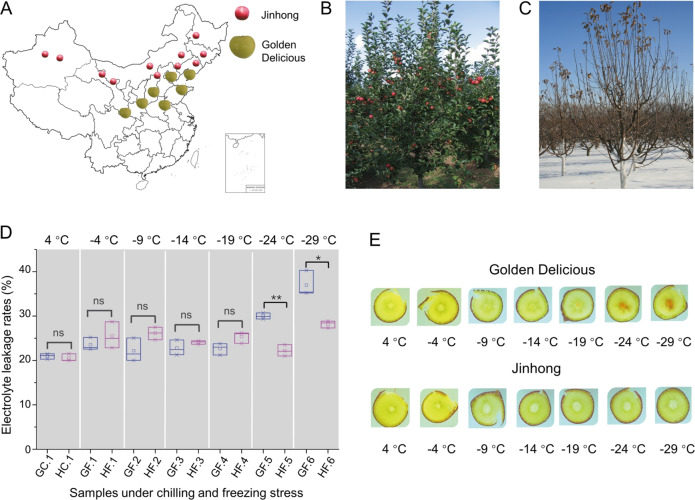
Cultivation distribution of “Golden Delicious” (G) and “Jinhong” (H) in China, “H” apple trees in the fruiting stage and dormancy, and electrolyte leakage rates and cross-section photos of 1-year-old branches of “G” and “H” stressed for 24 h at seven low temperatures. **A** Distribution of “G” and “H” cultivation in China; **B**, **C** “H” apple trees in the fruiting stage and dormancy in winter; **D** Electrolyte leakage rates (ELRs) of 1-year-old branches of “G” and “H.” GC.1 and HC.1, 4 °C/24h chilling treatments for “G” and “H”; GF.1 and HF.1, −4 °C/24h freezing treatments for “G” and “H”; GF.2 and HF.2, −9 °C/24h; GF.3 and HF.3, −14 °C/24h; GF.4 and HF.4, −19 °C/24h; GF.5 and HF.5, −24 °C/24h; GF.6 and HF.6, −29 °C/24h. “ns” indicates *p* > 0.05; “*”, *p* ≤ 0.05; “**”, *p* ≤ 0.01. **E** Analysis of cross-sections of 1-year-old branches of “G” and “H” that were treated for 24h at seven low temperatures

“Jinhong” (hereafter “H”) is one of the primary apple cultivars in cold areas in China, and it has better cold resistance than its male parent, “Golden Delicious” (hereafter “G”)^35^ (Fig. 1A–C). Its female parent, “Hong Taiping” [*M. prunifolia* (Willd.) Borkh.], contributes more to the freezing tolerance of “H.” Thus, “H” apple trees can resist freezing stress and damage at temperatures as low as −30 °C in their cultivation regions, whereas “G” trees suffer serious freezing injury and death at similar temperatures^36^. The cold tolerance of apple tree bark is important for their survival in dormancy^37^. Generally, the living bark cells of many woody species are considerably hardier in midwinter than the living xylem cells^38^, whereas the central pith tissue of the branch is more sensitive to freezing injury than the xylem tissue^37^. The anomalous color changes of the pith and xylem of branches in winter are used to indicate the freezing stress level of the branches. Although bark plays an indispensable role in keeping branches alive under freezing stress, transcriptomic profiling to investigate bark freezing tolerance in apple in different phases of stress between chilling and freezing temperatures has been limited. In this study, bark from 1-year-old branches of “G” and “H” trees was used for transcriptomic research. To clarify the gene regulation and expression patterns of apple branch bark in dormancy under cold stress from 4 to −29 °C, we adopted the WGCNA method^27^ to analyze the transcriptome data. These analyses can contribute to the understanding of freezing tolerance in apple and provide important insights into the molecular networks underlying the cold tolerance of apple branch bark.

## Results

### Transcriptomes of 1-year-old branches from “Golden Delicious” and “Jinhong” under cold treatments

The electrolyte leakage rates (ELR) is one of the frequently used parameters for evaluating plant cold resistance because it reveals the changes in membrane permeability caused by cold stress^39^. To detect the differences in cold tolerance between “Golden Delicious” (“G”) and “Jinhong” (“H”) (Fig. 1A–C), we estimated the ELRs of 1-year-old branches of “G” and “H” that were treated for 24 h at seven low temperatures, from 4 to −29 °C (Fig. 1D) (see the “Materials and Methods” section). The ELR of “G” increased as the temperature was reduced from −19 to −24 °C and to lower temperatures. This observation is consistent with the color changes in the pith and xylem of the branch (Fig. 1E). In contrast, the ELR of “H” remained at ~24% until the temperature decreased to −29 °C. The results also showed that the ELR of “G” was similar to that of “H” at 4, −4, −9, −14, and −19 °C; however, the ELRs of the two cultivars were significantly different at both −24 and −29 °C. These results indicate the difference in freezing tolerance between “G” and “H”; low-temperature damage to cell membranes occurred in the 1-year-old “G” branch at −19 °C/24 h, resulting in necrosis in the pith tissue^40^. However, “H” responded differently from “G” to the low-temperature treatments. The pith of the branches of “H” did not exhibit any distinct vascular tissue browning when exposed to even lower temperatures (Fig. 1E). Compared with “G”, “H” was likely better at protecting its tissues and maintaining cell membrane homeostasis at −19, −24, and −29 °C. As an indicator of the difference in freezing tolerance between the two genotypes, the browning at the center of the branches was a visible indicator of the stressed states of the branches. In this study, we mainly focus on the correlation between the electrolyte leakage rates in branches at low temperatures with gene expression.

Here, we classified the six freezing stress treatments into two phases: the early freezing stress phase (−4, −9, and −14 °C) (Phase I) and the late freezing stress phase (−19, −24, and −29 °C) (Phase II). These divisions were made according to the treatment temperatures and observations of the ELR results in the branches and the browning in the pith tissues. In Phase I, the ELR values showed nonsignificant variation, and the branches were likely tolerant of the relatively mild freezing stress. In Phase II, ELR values increased gradually, and the branches had to withstand freezing injury at the much lower temperatures. Therefore, studies of gene expression patterns and regulatory networks in Phase I and Phase II could facilitate an analysis of the differences between “G” and “H” in their transcriptomic changes related to bark freezing tolerance.

**Fig. 2:**
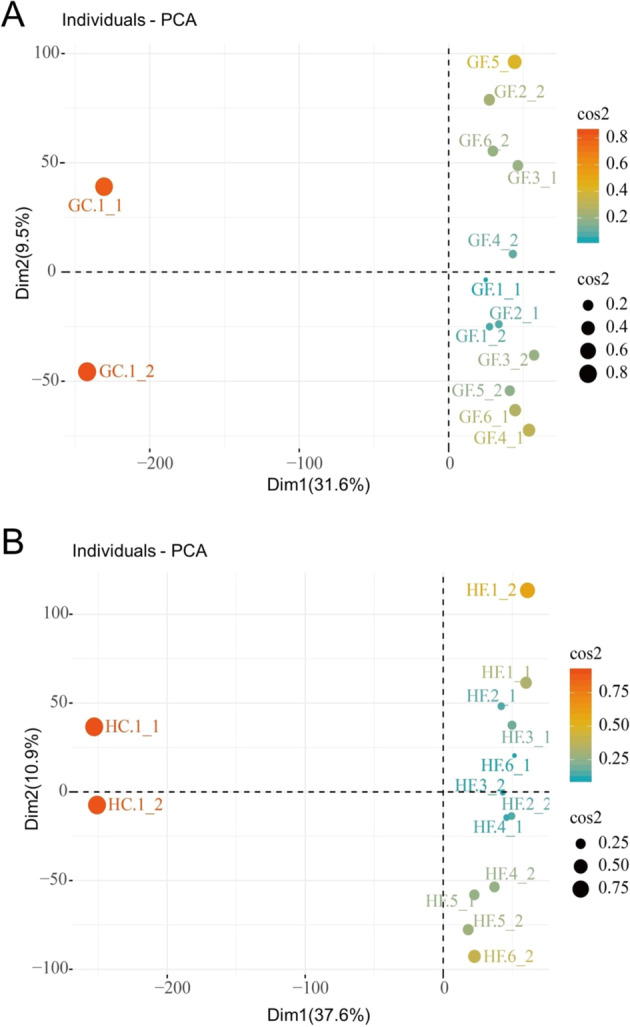
Principal component analysis (PCA) of expressed genes (FPKM ≥ 0.01) in “Golden Delicious” (G) and “Jinhong” (H) apple. **A** PCA results of 32,025 expressed genes in “G”. **B** PCA results of 30,745 expressed genes in “H”. GC and HC represent the chilling treatments applied to “G” and “H”; GF and HF represent the freezing treatments applied to “G” and “H.” The cos^2^ values (square cosine, squared coordinates) represent the quality of the variable representations on the factor map. A high cos^2^ value indicates a good representation of the variable on the principal component axis

Approximately 693 million clean paired-end reads with 150 bp in length were generated from the RNA-sequencing transcriptomes of the 28 samples. On average, 80.40% and 75.55% of the total clean reads from “G” and “H” apples, respectively, were uniquely mapped to the *M. domestica* genome^35^ (Table S1). Additionally, 32,025 and 30,745 genes were detected as expressed genes (FPKM ≥ 0.01) in “G” and “H”, respectively, and these genes accounted for more than 48% of the transcripts of the entire apple genome (63,517 transcripts). Principal component analysis (PCA) was used to visualize the overall changes in expressed gene data (FPKM ≥ 0.01) in response to the chilling and freezing stress. The first principal components of the gene expression data in “G” and “H” explained 31.6% and 37.6% of the total variance of the expressed genes, respectively (Fig. 2). The principal components of the gene expression data were divided into two groups along the first principal component axis according to chilling stress and freezing stress in both “G” and “H” and revealed the potential different specific gene functions of those two groups of genes. Furthermore, the first principal components of the differentially expressed genes (DEGs) in “G” and “H” explained 68.7% and 70.8% of their total variances, respectively (Supplementary Fig. S2). The principal components of DEG expression between “G” and “H” could be divided into four groups, indicating different gene expression patterns among them (Supplementary Fig. S3).

We also identified 4173 (G) and 7734 (H) DEGs in response to low-temperature stress between the chilling and freezing treatment samples, representing 6.57% and 12.18% of the whole-genome transcripts (Supplementary Fig. S1). In Supplementary Fig. S1, we use GF.1, GF.2, GF.3, GF.4, GF.5, and GF.6 to represent samples of “G” under freezing stress at −4, −9, −14, −19, −24, and −29 °C; and HF.1, HF.2, HF.3, HF.4, HF.5, and HF.6 represent samples of “H” under freezing stress at −4, −9, −14, −19, −24, and −29 °C. Supplementary Fig. S1 shows the 867 upregulated DEGs (Supplementary Fig. S1A (a)) and 722 downregulated (Supplementary Fig. S1B (a)) DEGs in “G” and the 1399 upregulated DEGs (Supplementary Fig. S1C (a)) and 2353 downregulated DEGs (Supplementary Fig. S1D (a)) in “H” in response to both Phase I and Phase II freezing stress. Hereafter, “Phase I–II” represents a differential expression pattern in response to the six freezing stress temperatures in Phase I and Phase II. A gene set from “G” that contains 258 upregulated DEGs and 270 downregulated DEGs only from GF.4, GF.5, or GF.6 (Supplementary Fig. S1A (b) and B (b)) but not from GF.1, GF.2 or GF.3 responds mainly to Phase II freezing stress; similarly, a gene set from “H” that includes 639 upregulated DEGs and 539 downregulated DEGs only from HF.4, HF.5, or HF.6 (Supplementary Fig. S1C (b) and D (b)) but not from HF.1, HF.2 or HF.3, responds mainly to Phase II freezing stress. Hereafter, “Phase II” represents a differential expression pattern in response to the freezing stress in Phase II. Moreover, 1143 upregulated DEGs and 1,183 downregulated DEGs in “G” (Supplementary Fig. S1A and B) were from GF.1, GF.2, or GF.3, but they were not DEGs in “Phase I–II”. Similarly, 2445 upregulated DEGs and 2001 downregulated DEGs in “H” (Supplementary Fig. S1C and D) were from HF.1, HF.2, or HF.3, but they were not DEGs in “Phase I–II”. Hereafter, we use “Phase I/II” to represent a differential expression pattern in which DEGs were activated at Phase I and only responded to some of the freezing stress temperatures.

### Weighted correlation network analysis (WGCNA) of the DEGs

**Fig. 3:**
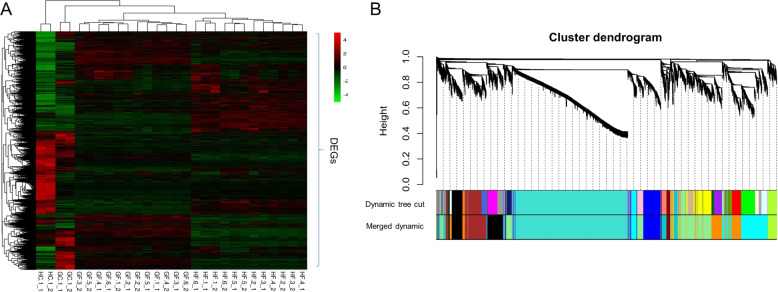
Expression heatmap of differentially expressed genes (DEGs) and hierarchical cluster dendrogram of gene coexpression modules of DEGs using the weighted gene correlation network analysis (WGCNA) method. **A** Expression heatmap of DEGs. The rows represent 9532 DEGs in “Golden Delicious” (G) and “Jinhong” (H). The columns indicate 28 stress treatment samples. Red indicates upregulated genes, and green indicates downregulated genes. GC and HC represent the chilling treatments applied to “G” and “H”; GF and HF represent the freezing treatments applied to “G” and “H”. **B** Hierarchical cluster dendrogram showing gene coexpression modules of DEGs

**Fig. 4:**
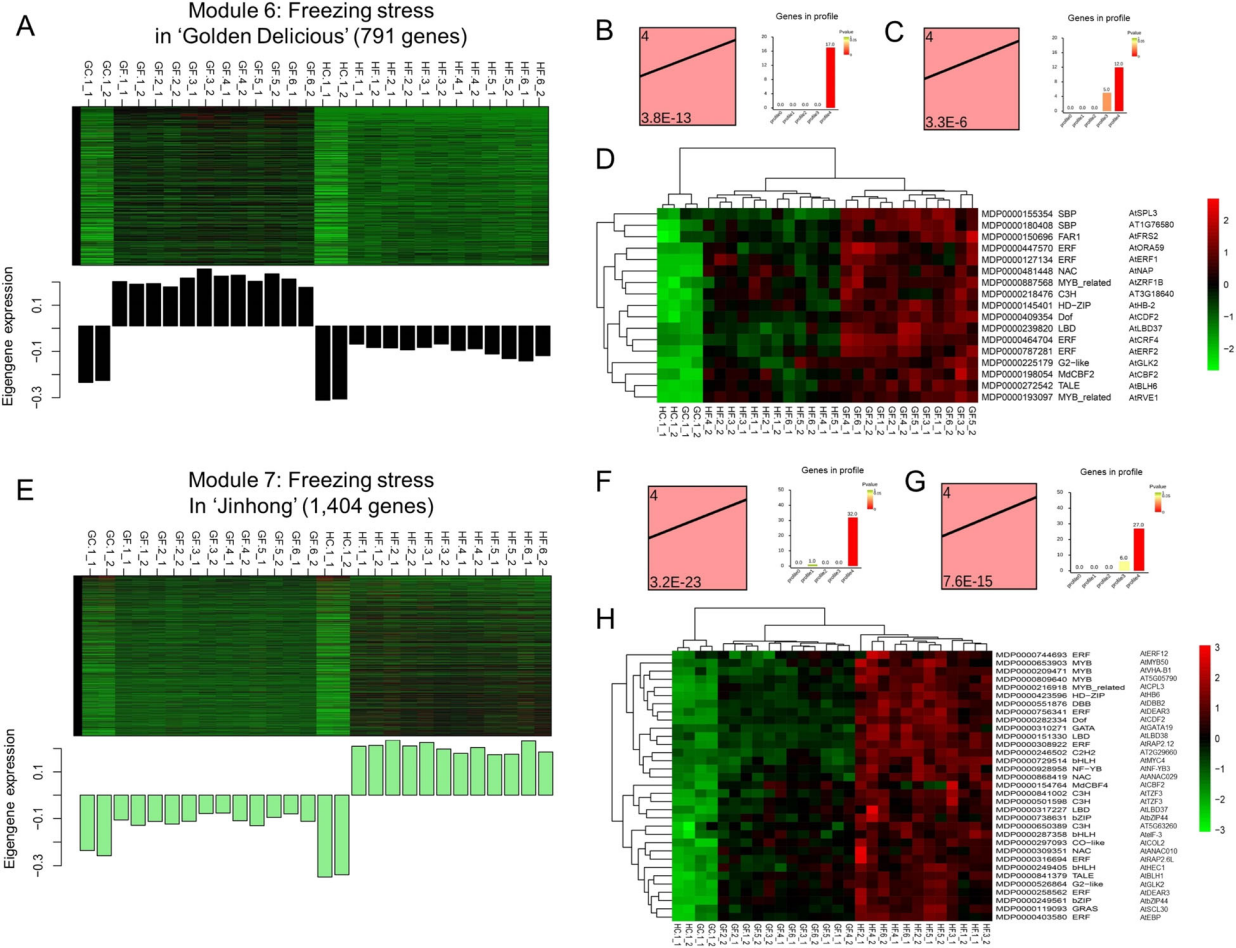
Expression heatmaps and profiles of differentially expressed genes (DEGs), eigengenes and hub transcription factors in modules 6 and 7. **A** and **E** Expression heatmaps and profiles of DEGs and eigengenes in modules 6 and 7 in response to freezing stress in “Golden Delicious” (G) and “Jinhong” (H), respectively. In the heatmap, rows represent the module genes, and columns indicate the stress treatment samples. Red indicates upregulated genes, and green indicates downregulated genes. GC and HC represent the chilling treatments applied to “G” and “H”; GF and HF represent the freezing treatments applied to “G” and “H”. The eigengene expression in WGCNA is defined as the first principal component of a given module, which can be representative of the gene expression profiles in a module. The bar graph of eigengene expression shows the eigengene value variance calculated from the singular value composition for each module. **B**–**C** and **F**–**G** The expression profile statistics and trends of hub transcription factors in modules 6 (**B**–**C**) and 7 (**F**–**G**) of “G” and “H”, respectively. The trend diagrams on the left show the most significant gene expression trends, including the gene expression profile number on the top left and its *p-*value on the bottom left; the bar graphs on the right indicate the gene number assigned to each gene expression profile, and the color reveals the *p*-value of the gene assignment significance. **D** and **H** The expression heatmaps of hub transcription factors in modules 6 and 7. The columns represent the 28 samples in this study. The rows show the hub transcription factors. Here, the transcription factor types and their homologs (gene locus ID or name) in *Arabidopsis thaliana* are also provided here

To classify the coexpression modules and identify intramodular hub genes based on gene expression data, a weighted correlation network was constructed using 9532 DEGs in “G” and “H” (Fig. 3A). In this study, a thresholding power of 8 was selected, which was the lowest power for a proper fit of the scale-free topology index, and WGCNA was performed on the 9532 DEGs of “G” and “H” identified above (Supplementary Fig. S4). Eleven coexpression modules were identified through the merged dynamic analysis of WGCNA (Fig. 3B and Supplementary Table S2), including module 1 (cyan, 1,070 genes), module 2 (turquoise, 3346 genes), module 3 (dark orange, 859 genes), module 4 (brown, 686 genes), module 5 (blue, 641 genes), module 6 (black, 791 genes), module 7 (light green, 1404 genes), module 8 (green yellow, 240 genes), module 9 (dark red, 131 genes), module 10 (dark turquoise, 284 genes), and module 11 (orange, 70 genes). Ten genes could not be placed in any of the modules and were initially assigned to the gray module. Of the eleven modules, seven modules are biologically meaningful because the eigengenes assigned to the modules are associated with the responses to low-temperature stress treatments in “G” and/or “H.” The eigengene roles were revealed as follows: the eigengene of module 1 corresponded to the chilling stress treatment response in “G” (Supplementary Fig. S5A); the eigengene of module 2, to the chilling response in “H” (Supplementary Fig. S5B); the eigengene of module 3, to the chilling and freezing responses in “G” (Supplementary Fig. S5C); the eigengene of module 4, to the chilling and freezing responses in “H” (Supplementary Fig. S5D); the eigengene of module 5, to the freezing responses in “G” and “H” (Supplementary Fig. S5E); the eigengene of module 6, to the freezing response in “G” (Fig. 4A); and the eigengene of module 7, to the freezing response in “H” (Fig. 4E).

The biological functions of the eigengenes in each module were further analyzed by investigating the relationships of the module eigengenes with low temperature (LT), accumulated low temperature (Ac_LT), and ELR (Supplementary Fig. S6). The correlation coefficients varied widely, from −0.5 to 0.65 in LT and Ac_LT and from −0.33 to 0.41 in ELR. The eigengenes of modules 1, 2, 3, and 4 were negatively correlated with LT and Ac_LT, especially in module 1 (*p* < 0.006) and module 2 (*p* < 0.01), suggesting that these four modules were mainly related to the chilling stress treatments. However, the eigengenes of modules 5, 6, and 7 were positively correlated (*p* < 0.05) with LT and Ac_LT, indicating that they were primarily associated with the freezing stress treatments rather than the chilling stress treatments. Therefore, the observed differences in gene expression in response to chilling and freezing stress were well supported by the eigengene analyses. These differences suggested the existence of not only specific gene sets responding to chilling or freezing stress but also many differences in specific gene sets between “G” and “H.”

**Fig. 5:**
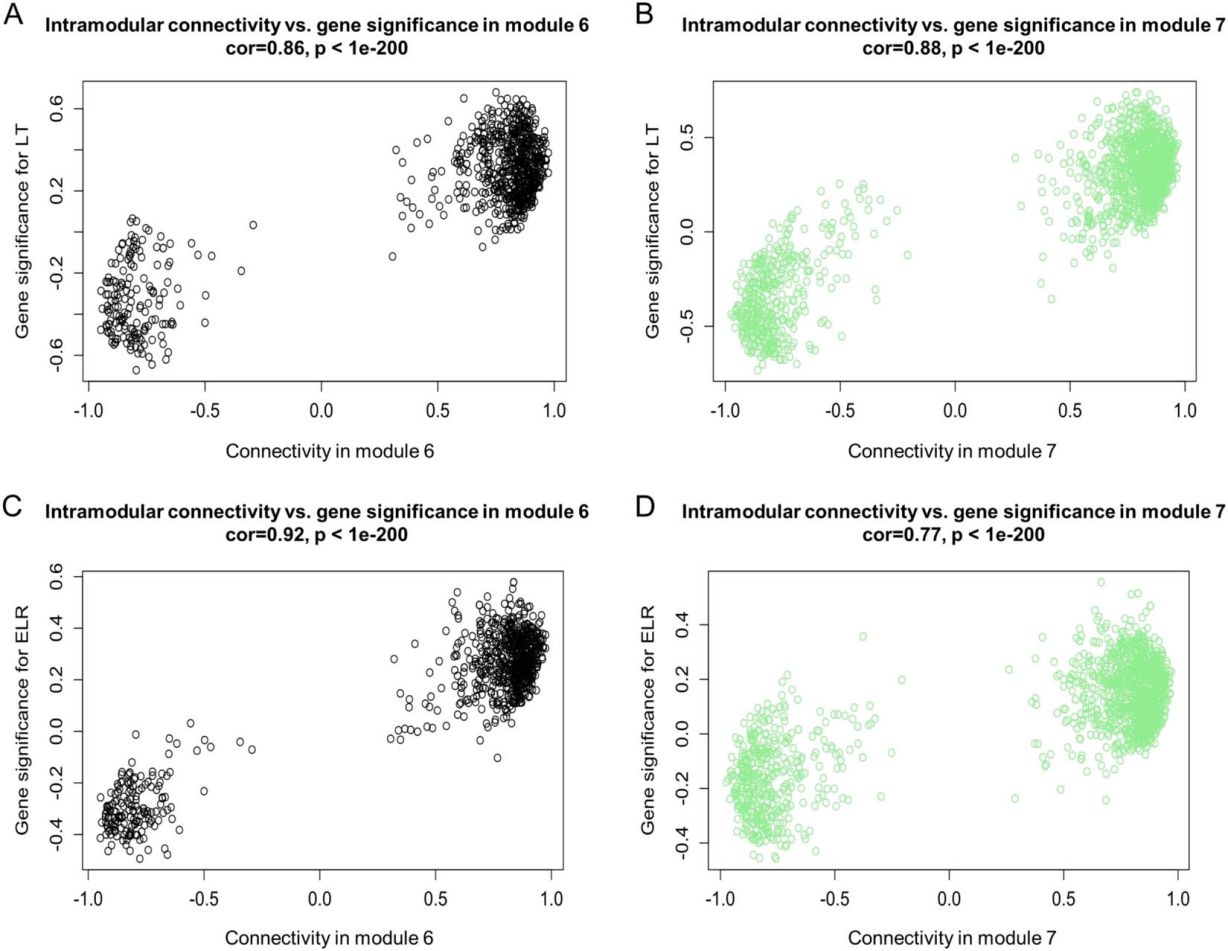
The gene significance for low temperature (*y*-axis) and electrolytic leakage rate (*y*-axis) vs. the intramodular connectivity (*x*-axis) plotted separately in modules 6 and 7. **A**, **B** Gene significance (GS) for low temperature (LT) vs. intramodular connectivity in modules 6 and 7. **C**, **D** GS for electrolytic leakage rate (ELR) vs. intramodular connectivity in modules 6 and 7. GS for LT or ELR means the low temperature-based GS or electrolyte leakage rate-based GS obtained by correlating the modules to LT or ELR values with the eigengene network methodology. Intramodular connectivity (*K*_ME_) is the module eigengene-based network connectivity

To identify cold tolerance-related hub genes by combining GS and module membership (intramodular connectivity) in a systems biology-based screening method, the module eigengene-based connectivity (intramodular connectivity) was calculated for each gene, and the GS of LT and ELR were plotted against the intramodular connectivity^27^ (Fig. 5 and Supplementary Figs. S7 and S8, Supplementary Table S2). In response to freezing stress, genes in modules 5, 6, and 7 had positive correlation coefficients. In contrast, modules 1, 2, 3, and 4 had negative correlations between GSs and intramodular connectivity values with regard to mainly chilling stress. Interestingly, each module eigengene could be divided into two groups, and this division was used to identify the hub genes of modules related to cold tolerance through the correlation between intramodular connectivity and GS. The hub genes related to freezing tolerance in modules 5, 6, and 7 showed highly positive correlations between intramodular connectivity and GS, whereas the hub genes related to chilling resistance in modules 1, 2, 3, and 4 showed strong negative correlations between intramodular connectivity and GS.

### Identification of hub genes and their expression profiles

In this study, 173, 598, 144, 111, 118, 126, and 230 hub genes were identified in modules 1, 2, 3, 4, 5, 6, and 7, respectively (Table S3). GO annotation was performed for these hub genes (Table S4). Among the seven modules, the genes in modules 6 and 7 responded specifically to freezing stress in “G” or “H.” As CORs are strongly dependent on TF regulation to increase freezing tolerance in plants^41^, we further analyzed the expression profiles of both the hub TFs in modules 6 and/or 7 and their homologs in Arabidopsis (Table S5). A total of 17 and 33 hub TFs were detected in modules 6 and 7, respectively.

These TFs were classified into 20 TF families (Table S3). Most of these families were CBF-related gene families, such as ERF, bHLH, bZIP, C3H, NAC, and GATA, in Arabidopsis^7,12,16^. In general, regardless of which module the hub TFs belonged to, they were upregulated during Phases I and II of freezing stress compared to their expression under chilling stress in each cultivar (Fig. 4A–G). However, the gene expression patterns of “G” and “H” in response to freezing stress in module 6 and module 7 were considerably different. For example, 33 hub TFs in module 7 had higher expression levels in “H” than in “G” under freezing stress in Phases I and II, which would account for the freezing tolerance of “H” (Fig. 4E and H). Seventeen hub TFs in module 6 had similar expression profiles and were responsible for cold resistance in “G” (Fig. 4D). Consequently, the larger number of hub TFs in module 7 and their higher expression levels suggest a more robust gene regulation capacity in “H” than in “G” that allows “H” to survive in colder regions. The above results suggest that the hub genes in the tolerant cultivar that were activated in the early phase of freezing stress may play key roles in all mechanisms of freezing tolerance. Furthermore, the transcription regulation differences present in the early and late freezing stress phases might result in “G” being sensitive to and “H” being tolerant of low temperatures. These results provide some useful suggestions for further study of the functions of hub TFs in apple bark.

### Hub TFs and their homologs related to CBF-dependent or CBF-independent pathways

Members of nine gene families, namely, ERF, C3H, G2-like, NAC, HD-ZIP, TALE, LBD, Dof, and MYB_related, were identified in modules 6 and 7. Members of the ERF gene family were the first to be analyzed, and *CBF* genes played key roles in cold tolerance^7^. Two *CBF* genes were defined as hub genes: *MdCBF2* (*MDP0000154764*)^6,42^ (intramodular connectivity, *K*_ME_, 0.77; GS_LT, 0.45) in module 6 and *MdCBF4* (*MDP0000198054*)^6,43^ (*K*_ME_, 0.57; GS_LT, 0.35) in module 7. *MdCBF2*, as a hub gene in module 6, responded in the same way to Phases I and II of freezing stress (Table S3_6). However, *MdCBF4* in module 7 mainly responded to the early phase of freezing stress in “H” (Table S3_7). *MdNAC029* (“Phase I–II”) in module 6 had similar intramodular connectivity and GS_LT values with *MdCBF2* (Table S3_6) and was negatively regulated by the transcription of *MdCBF*s^3^. In module 7, there were two hub ERF TFs whose homologs in Arabidopsis were related to the CBF pathway: *AtDEAR3* (*AT2G23340*, homolog of *MDP0000756341*, “Phase I–II”; 1.14E^−53^) (Table S3_7), which is regulated by *AtCBF1*, *AtCBF2*, and *AtCBF3*^7^ (Table S5), and *AT5G67190* (homolog of *MDP0000258562*, “Phase I–II”; 9.5E^−48^), which can be activated by *CBF*s^13^. Moreover, *AT1G72360* (homolog of *MDP0000308922*, “Phase I–II”; 3.59E^−40^) was downregulated in *cbf1* or *cbf3* mutants^16^, and *AT5G13330* (homolog of *MDP0000316694*, “Phase I–II”; 9.71E^−47^) was expressed differently in *cbf1/cbf2/cbf3* triple mutants (*cbf123*)^13^. *AtEBP* (*AT3G16770*, homolog of *MDP0000403580*, “Phase I–II”; 2.82E^−38^) was regulated by *AtZAT10* and *AtZF*^7^. Second, of the members of the other eight hub TF families shared by modules 6 and 7, only four homologs in Arabidopsis were regulated by *CBF*s in members of the MYB-related, G2-like, and C3H families. For example, *AT4G01060* (homolog of *MDP0000216918*, “Phase I–II”; 1.31E^−15^; MYB_related) was upregulated by *AtCBF1* and *AtCBF3*^16^, and *AT5G63260* (homolog of *MDP0000650389*, “Phase I–II”; 5.32E^−93^; C3H) was upregulated by *AtCBF1*, *AtCBF2*, and *AtCBF3*^16^ (Table S5). Nevertheless, the six other homologs in members of the TALE, NAC, LBD, HD-ZIP, and Dof families in Arabidopsis, were not correlated with *AtCBF*s or regulated by non-CBF TFs. For instance, *AT1G69490* (homolog of *MDP0000868419*, “Phase I–II”; 1.47E^−80^; NAC) and *AT5G39660* (homolog of *MDP0000282334*, “Phase I–II”; 4.78E^−100^; Dof) were differentially expressed in *cbf123* (Table S5).

Fourteen other hub TFs were detected only in module 7, and they belonged to nine TF families, namely, NF-YB, MYB, GATA, GRAS, DBB, CO-like, bZIP, bHLH, and C2H2. Among them, Arabidopsis homologs of three apple TFs (GRAS, DBB, and bHLH) were related to CBF regulation. *AT3G46600* (*MDP0000119093*, “Phase I–II”; 6.1E^−150^; GRAS) was downregulated by *AtCBF1*, *AtCBF2*, and *AtCBF3*^16^ (Table S5). *AT4G39070* (homolog of *MdBB X21*, “Phase I–II”; 4.55E^−56^; DBB)^44^ and *AT5G67060* (homolog of *MDP0000249405*, “Phase I–II”; 7.79E^−50^; bHLH) were activated by *CBF*s after the low-temperature treatments^13^ (Table S5). Arabidopsis homologs of seven apple TFs were also involved in the CBF-independent pathway under low-temperature stress. For example, *AtNF-YB3* (*AT4G14540*, homolog of *MDP0000928958*, “Phase I–II”; 2.05E^−68^; NF-YB) was differentially expressed in *cbf123*, and *AtbZIP44* (*AT1G75390*, homolog of *MdbZIP48*^45^, “Phase I–II”; 1.39E^−17^; bZIP) was regulated by *HSFC*^17^ (Table S5). On the other hand, three hub TFs from the SBP and FAR1 families were detected only in module 6. Two Arabidopsis homologs, *AT1G76580* (homolog of *MDP0000180408*, Phase I/II; 3.68E^−62^; SBP) and *AT2G32250* (homolog of *MDP0000150696*, Phase I-/II);3.24E^−106^; FAR1), were regulated by *CBF*s^13,16,17^ (Table S5).

Therefore, previous functional studies of Arabidopsis homologs suggested the potential roles of these apple hub genes in CBF-independent pathways. Many hub TFs with higher intramodular connectivity or GS than *MdCBF2* or *MdCBF4* showed close correlations with freezing stress in the molecular network.

### Hub TFs and CORs in response to more severe freezing stress

We screened the DEGs that specifically responded to more severe freezing stress treatments (Phase II) at −19, −24, and −29 °C compared to the chilling stress treatment at 4 °C (Supplementary Fig. S1A (b), B (b), C (b), and D (b)), through cluster analyses of the DEGs expressed in the treated samples. Furthermore, we identified the hub genes that specifically responded to Phase II freezing stress. In module 7, two hub TFs and 14 CORs responded specifically to Phase II freezing stress in “H” (Table S3_7). One of the hub TFs, *MDP0000744693*, has an AP2 domain and is a homolog of *AT1G28360* (*E*-value = 2.29E^−31^). *AT1G28360* is expressed differentially in response to cold stress and in *cbf123*^13^. One of the hub CORs, *MDP0000836119*, encodes a protein with a 2Fe-2S iron-sulfur cluster binding domain (Fer2)^46^ and is a homolog of *AT1G10960* (*E*-value = 1.56E^−42^) (Table S5). *AT1G10960* is differentially expressed in response to cold stress in *Arabidopsis*. Similar proteins are involved in modulating oxidoreductase activity; for example, the tomato Fer2 protein is important for mediating reactive oxygen species signaling^46^. Two other hub CORs, *MDP0000348470* and *MDP0000348471*, encode proteins with annotated C2 domains for Ca^2+^-binding^47^. C2 domains can interact with cellular membranes and are involved in membrane trafficking, GTPase activation and other processes^47^. *MDP0000150999*, a hub gene, contains an annotated N-terminal domain of xylanase inhibitor (TAXi_N). TAXi_N proteins can inhibit xylanase through proteolysis, which is vital for preventing plant cell wall degradation^48^.

Among the hub TFs and CORs in module 6, six hub CORs were induced in response to Phase II freezing stress in “G” (Table S3_6). Two of these hub CORs, *MDP0000259640* and *MDP0000785413*, were annotated with “extracellular region” (GO:0005576) and “plant-type cell wall organization” (GO:0009664) and had a possible role in the degradation of cellulose and pectin-containing cell walls^49^. Another hub COR, *MDP0000530457*, has a Cupin_1 domain^50^. Members of the cupin superfamily are involved in cell wall synthesis and are induced in response to abiotic stress, such as desiccation or starvation^50^. These results suggest that increased expression at the transcript level was probably important for the genes that encode proteins located in the extracellular space outside the plasma membrane, as these proteins were part of the stress response after the significant electrolyte leakages in Phase II in “G.”

### Hub CORs responding to water and related to oxidoreductase activity

In general, freezing damage is not a consequence of low temperatures but rather the result of cellular dehydration generated by extracellular ice formation^8^. Interestingly, hub genes that were enriched for controlling the response to water (GO:0009415; *p* = 5.23E^-8^) were detected only in module 7 (Table S4). Ten genes were involved in this process and accounted for 29.41% of the annotated water-responsive genes in the background datasets of the apple genome (Table S4). The expression profiles of hub genes in different low-temperature treatments of “H” and “G” samples showed that these genes were upregulated more during freezing stress than during chilling stress (Supplementary Fig. S9A and B). Four of these ten hub genes in module 7 responded to water, were expressed at higher levels in “H” under freezing stress from −4 to −29 °C and were responsible for resisting cellular dehydration in “H” (Supplementary Fig. S9C). Moreover, two of the four hub genes in module 7 were identified as dehydrin genes in apple: *MdDHN3* (*MDP0000689622*, “Phase I–II”) and *MdDHN4* (*MDP0000360414*, “Phase I–II”)^51^ (Supplementary Fig. S9C and Supplementary Table S3_7). Dehydrin genes in plants prevent cell dehydration in the bark and xylem at low temperatures^5^. In Arabidopsis, *AtERD10* and *AtCOR47* are homologs of *MdDHN3* (*E*-value = 5.62E^−13^) and *MdDHN4* (*E*-value = 6.29E^−12^), respectively, and the overexpression of *AtERD10* or *AtCOR47* enhances the freezing tolerance of Arabidopsis^19^. Nevertheless, the other two hub genes, *MDP0000360672* (“Phase I–II”) and *MDP0000268941*(“Phase I–II”), did not have any detected protein domain in Pfam^52^ or homologs in Arabidopsis (Table S6). These water-responsive hub genes are highly expressed in module 7 and may improve the freezing tolerance of “H”.

Reactive oxygen species (ROS), including hydrogen peroxide, superoxide anions, hydroxyl radicals, and singlet oxygen, accumulate significantly under abiotic stress conditions and result in oxidative damage and cell death^53^. According to the hub gene GO annotations (Table S4), 5 and 15 oxidoreductase activity-related GO terms were detected in 9 and 17 hub genes from modules 6 and 7, respectively (GO:0016491; *p* = 0.16 in module 6; *p* = 0.67 in module 7). Most Arabidopsis homologs of these hub genes exhibited significant associations with low-temperature stress. Among these GO annotations, two types of oxidoreductase activities were present in both modules, which acted either on the “paired donors, with incorporation or reduction of molecular oxygen”, or “CH-OH group of donors.” Moreover, four kinds of oxidoreductase activities of hub gene GO annotations acted on the donors of “aldehyde or oxo group”, “CH-NH group”, “sulfur group”, and “diphenol-related substances”, which were annotated only in module 7. Arabidopsis homologs of these hub genes that were related to cold acclimation were detected. For instance, *AT3G04120* (homolog of *MDP0000288010*, “Phase I–II”; *E*-value = 0) and *AT3G62260* (homolog of *MDP0000295277*, “Phase I–II”; 9.61E^−127^) are DEGs in *cbf123* that respond to cold stress^13^, and *AT3G24170* (homolog of *MDP0000897124*, “Phase I/II”; 1.47E^−51^) is upregulated by *AtCBF1*^16^ (Table S6). Moreover, *MDP0000555589* (“Phase I–II”) has a conserved polyphenol oxidase (PPO) domain (PPO1_KFDV), and the expression of *PPO* genes was detected in stem tissues, especially in xylem, xylem parenchyma, pith tissues, and cells in vascular bundles^54^. The specific expression characteristics of PPO genes are essential to consider in studying freezing damage and necrosis in the xylem, pith, and vascular tissues of apple branches.

### DEGs identified between the five colder freezing stress treatments and the least-cold freezing stress treatment and their functional analysis

To compare the transcriptomic differences between the different freezing treatments, we analyzed the differences between the expression of each detected expressed gene (FPKM ≥ 0.01) at the five lower freezing temperatures (−9, −14, −19, −24, and −29 °C) and that at −4 °C. We identified 111 DEGs in “G” (Supplementary Fig. S10) and 222 DEGs in “H.” Then, we analyzed the important genes that responded specifically to Phase II (at −19, −24, and −29 °C).

Based on the GO enrichment analysis of these DEGs, we found that some of the DEGs were closely related to methionine biosynthetic process (GO:0071266; *p* = 0.0008; GO:0006556; *p* = 0.003), carbohydrate biosynthetic process (GO:0016051; *p* = 0.04), and proton transmembrane transport (GO:1902600; *p* = 0.03) (Table S7). Methionine can protect the cell membrane from oxidative stress and has been connected with the pentose phosphate pathway in genetic experiments^55^. In the tolerant cultivar (“H”), for example, *MDP0000210722*, which is related to the S-adenosylmethionine biosynthetic process, was activated early at −19 °C, and its homolog in Arabidopsis, *AT2G36880* (*E*-value = 3E^−49^), is methionine adenosyl-transferase 3 (Table S8). Nevertheless, in the sensitive cultivar, “G”, *MDP0000889709*, which is also involved in the methionine biosynthetic process, was activated when the temperature decreased to −29 °C. Moreover, the number of upregulated DEGs at −24 °C was significantly different between “G” and “H” (Supplementary Fig. S10). Among the 24 DEGs in “H”, *MDP0000154144* was related to proton transmembrane transport (GO:1902600; *p* = 0.03), and its homolog in Arabidopsis, *AT4G34720*, exhibits proton-transporting ATPase activity (Tables S7 and S8); *MDP0000686454* and *MDP0000859430* might be involved in protein ubiquitin functions because the homolog of *MDP0000686454* in Arabidopsis (*AT4G24210*; *E*-value = 1E^−58^) encodes an F-box protein as a component of the E3 ubiquitin complex; and the homolog of *MDP0000859430* in Arabidopsis (*AT1G02860*; *E*-value = 2E^−38^) encodes a likely ubiquitin E3 ligase with RING and SPX domains that is involved in mediating immune responses (Table S7 and Table S8).

To search for a possible relationship between the DEG number and DEG functions in the different freezing treatments, we compared the number of DEGs between the two cultivars at each temperature. The results showed more downregulated DEGs in “H” than in “G” at each of the three temperature points (−14, −19, and −24 °C; *p* < 0.001). In “G”, several downregulated DEGs were closely related to ATP synthesis-coupled electron transport (GO:0042773; *p* = 0.005), glycolytic process (GO:0006096; *p* = 0.03) and gluconeogenesis (GO:0006094; *p* = 0.005) in both the −14 and −19 °C treatments (Table S7). For instance, *AT3G52730*, a homolog of *MDP0000185086* in Arabidopsis, was involved in mitochondrial electron transport related to ATP synthesis (Table S8). *MDP0000793357* was downregulated in “G”, and its homolog in Arabidopsis (*AT5G42740*; *E*-value = 5E^−115^) was involved in gluconeogenesis and glycolysis (Table S8). Nevertheless, the downregulated DEGs in “H” were correlated with carbohydrate transport (GO:0008643; *p* = 0.02), lipid biosynthetic process (GO:0006629; *p* = 0.008), and negative regulation of transcription (GO:0045892; *p* = 0.03) at −14, −19, and −24 °C. For example, *MDP0000315959* and *MDP0000285074* in “H”, which were downregulated at −19 °C, were involved in lipid localization (GO:0010876; *p* = 0.03) and lipid transport (GO:0006869; *p* = 0.03). Interestingly, the homolog of one of the upregulated DEGs at −19 °C in “H” (*MDP0000932449*) in Arabidopsis, *AT4G12510* (*E*-value = 2E^−47^), is a bifunctional inhibitor of lipid-transfer proteins and hydrophobic proteins (Table S8). This result showed that some nonessential gene activities in the tolerant cultivar were probably suppressed in order to save energy and withstand the freezing stress.

### Expression pattern validation of hub genes in module 7

**Fig. 6:**
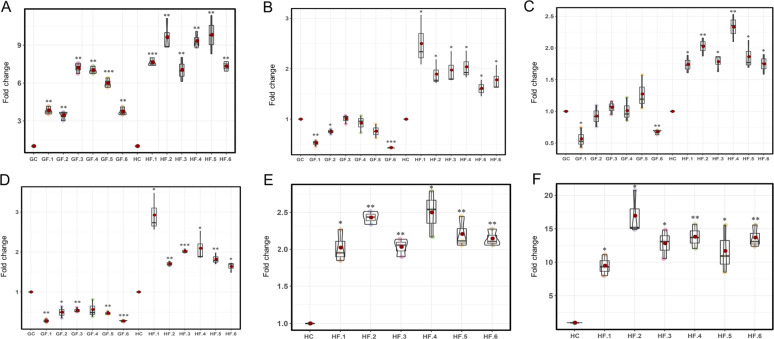
Gene expression patterns of six hub genes in module 7 in response to freezing stress in “Jinhong” apple determined by using real-time RT-PCR. **A**–**D** Expression patterns of *MDP0000308922*, *MDP0000291220*, *MDP0000451624*, and *MDP0000198451* in “Golden Delicious” (G) and “Jinhong” (H). **E**–**F** Expression patterns of *MdbZIP2* (*MDP0000249561*) and *MDP0000360672* in “H”. GC and HC represent the chilling treatments applied to “G” and “H”; GF and HF represent the freezing treatments applied to “G” and “H”. “*”, “**”, and “***” indicate significance at the 0.05, 0.01, and 0.001 levels according to the *t-*test for the difference in hub gene expression ratios between the samples in the chilling and freezing treatments.

To validate the hub gene expression patterns in response to a specific low-temperature process, the expression ratios of six high *K*_ME_ hub genes in module 7 in the “G” and “H” samples were tested using real-time reverse transcription PCR (RT-PCR). These hub genes were upregulated significantly (*p* < 0.05) in the samples of “H” subjected to the freezing treatment compared with their expression in “G” samples (Fig. 6). *MdbZIP2* (*MDP0000249561*)^45^ has a bZIP_1 protein domain, and its expression quantity was consistently high, ranging from 7.33- to 9.81-fold (*p* < 0.01) in the samples of “H” in the freezing treatment. The significant expression ratio of *MdbZIP2* was also determined to be 3.45- to 7.21-fold in “G” (Fig. 6A). In Arabidopsis, *ATGBF5* (*AT2G18160*), a homolog of *MdbZIP2* (*E*-value = 2.47E^−41^), partially mediates primary KIN10 signaling in response to stress^56^ (Table S9). *MDP0000291220*, *MDP0000451624*, and *MDP0000198451* responded to only the freezing treatment “H” samples (Fig. 6B–D), and their expression ratios were higher than those in the chilling treatment “H” samples. In contrast, these three hub genes did not show significant differences in their response to the chilling and freezing treatments in “G”. In Arabidopsis, *ATRD21A* (response to dehydration 21A; homolog of *MDP0000291220*; *E*-value = 2.04E^−159^) is a drought-inducible cysteine proteinase belonging to the papain family^57^ that is expressed in response to dehydration (Table S9). Moreover, two of six hub genes, *MDP0000308922* and *MDP0000360672*, were expressed only in “H” (Fig. 6E–F). One of these two genes, *MDP0000308922*, has an AP2 protein domain; its homolog, *ATHRE1* (*AT1G72360*) (*E*-value = 3.59E^−40^), is a hypoxia-inducible ethylene response factor that affects anaerobic responses in Arabidopsis^58^.

## Discussion

WGCNA, which provides a time series analysis of gene expression in response to cold stress, is an essential tool for determining the functions of genes related to freezing tolerance in apples or, more broadly, woody plants^27^. We identified modules of coexpressed genes related to these phenotypic gradients through an eigengene network methodology^27^ and revealed potential primary functional genes and pathway targets of cold tolerance^27,28^. These results could be a meaningful step toward a deeper understanding of the molecular mechanism of the phenotype-genotype map of cold tolerance. Furthermore, we avoided significant gene absences and provided highly meaningful information by comprehensively screening the hub genes. We increased the candidate hub gene ratio with genes in the top 20% of *K*_ME_^27^ or GS^27^, and we extracted hub genes based on *K*_ME_ as well as GS. Module membership and gene significance can be combined into a systems biological screening method for finding related target genes^27,33,34^.

Chilling and freezing tolerance take effect through different resistance mechanisms in plant tissues^41,59^. In woody plants, cold tolerance in bark tissues, including the cambium, cortex, and phloem, is essential for the plant to endure freezing temperatures during long winters. In this study, thousands of DEGs and their specific and dynamic behaviors between chilling stress (4 °C) and freezing stress (−4 to −29 °C) were observed in apple bark tissues; these observations revealed high freezing tolerance in the bark and the close relationship of freezing tolerance to gene expression. The first principal component^31,32^ of the differences in gene expression also showed the clear transition in gene behaviors between chilling and freezing in the bark cells. Furthermore, we found that most hub TFs in modules 6 and 7, such as *CBF*s, were activated from the start of freezing stress^5^. They maintained upregulated expression throughout the phases of freezing stress in “G” and “H”. In other words, gene transcriptional regulation in apples was sensitive to freezing stress rather than to chilling stress. The hub genes activated under early freezing stress may play key roles in overall freezing tolerance. The differences in gene behavior between “G” and “H” were clearly more related to early freezing stress than to extreme freezing temperatures^60^. “Jinhong” is a hybrid between its male parent, “Golden Delicious”, and its female parent, “Hong Taiping.” “Hong Taiping” has more cold tolerance than both “H” and “G.” The introduction of apple germplasm into this hybrid could contribute greatly to understanding the differences in gene expression and cold tolerance between “H” and “G.” Despite the germplasm differences between “H” and “G”, we have already identified some candidate genes that are probably related to cold tolerance in “H” but not in “G”. These genes were coexpressed with important known functional genes, such as *MdCBF2*, *MdCBF4*^6,25^, and *MdNAC029*^3^, and could shed more light on important candidate genes for further gene function studies.

Knowledge of gene functions under low-temperature stress in plants is primarily based on research on a few traditional model plants, such as Arabidopsis^16,17^ and rice^61^. These herbaceous species are not resistant to freezing damage under natural conditions. Therefore, it is impossible to obtain true gene expression data from these plants in response to freezing injury. In this work, module 7, which responded mainly in “H”, tended to exhibit more efficient transcription regulation and freezing-regulated gene expression than “G” because of its more interconnected hub TFs and COR genes. The molecular basis of the hub genes in module 7 provided stronger cold tolerance to “H” via some specific biological functions, including “response to water” (ten hub genes) and “oxidoreductase activity” (seventeen hub genes). Moreover, the polyphenol signaling system may be closely related to freezing tolerance in woody plants^54^. Polyphenol oxidase (PPO) expression has been confirmed in woody plants, specifically in the xylem and pith tissues and the cells in vascular bundles^54^. The PPO activity in the apple bark was measured in this work, which may have highlighted important candidate genes for studying freezing damage and necrosis in the xylem, pith and vascular tissues of apple branches.

In the last two decades, cold resistance research in plants has often focused on the CBF pathway. However, it has not been reported whether the CBF pathway is involved in freezing damage caused by temperatures as low as −24 to −29 °C. In our work, the presence of hub genes such as *MdCBF2* and *MdCBF4* in modules 6 and 7 suggests that these modules are related to the CBF pathway^41^ because coexpressed modules are usually involved in the same biological pathways^62^. The above results indicate that the CBF pathway was also involved in resistance to freezing damage at approximately −30 °C. However, the modules also contain many hub TFs with high *K*_ME_ and GS values that are likely to play important, but not fully understood, roles in cold tolerance functions. Some of these genes are likely independent of the CBF pathway. This study raises the possibility that transcription regulation in branch bark may involve many levels of an interconnected regulation network that are correlated with the different types of TFs. We also provide the alignment analysis of the apple genome and the DEGs in this study with CBF^41,63^, ABA^57^, BR^64^, and ETH^65–67^ pathway-related genes in Arabidopsis as a reference for further studies related to abiotic stress (Table S10–12).

Moreover, we also studied the DEGs between specifically the late freezing stress treatments at −19, −24, and −29 °C and the first freezing stress treatment at −4 °C. In the sensitive cultivar, we found that some important genes involved in ATP synthesis, glycolytic process and gluconeogenesis were downregulated starting at the beginning of the early freezing stress. Many nonessential gene activities in the tolerant cultivar were suppressed, which helped save cellular energy during the early and late freezing stress phases. Notably, the upregulated DEGs related to methionine biosynthetic process and carbohydrate biosynthetic process under late freezing stress in the tolerant cultivar are likely beneficial to homeostasis between cellular membranes and ROS^55,68^. This response partly explains the increasing ELRs in “G” when the temperature decreased to −19 °C and the significant differences between “G” and “H” at −24 °C and −29 °C (Fig. 1D).

Several potential limitations of this study are necessary to discuss here. This work represents a relatively new approach to low-temperature treatment, i.e., the use of a programmed low-temperature control system. However, the study did not include a light treatment, which prevents further detailed interpretation of circadian clock-regulated gene expression trends related to cold responses. We also noted that one hub MYB-related TF (*MDP0000193097*) in module 6 was homologous with a circadian clock-associated 1, *AtCCA1* (*AT2G46830*; *E*-value = 4.18E^−30^)^69^ (Table S6). This association suggests that circadian timing plays a certain role in freezing resistance. Second, the hub genes in modules 1, 2, 3, 4, and 5 did not undergo as detailed an analysis as those in modules 6 and 7. However, their biological meanings were provided through the coexpression network, and we obtained informative annotations for them, such as their conserved domain, GO, and homologous gene annotations. Our work may provide targets for subsequent studies of the differences in molecular mechanisms and functional genes between chilling and freezing tolerance. For example, many hub genes in modules 1 to 5 were related to defense responses (Table S4) and the identification of these specific hub genes linked defense responses to potential mechanisms in response to chilling stress. Third, we focused on the transcriptome data for the bark of a 1-year-old branch. Although the results might not fully explain the electrolyte leakage phenotypes at −24 °C and −29 °C, it is possible that other tissues in the branch that were not analyzed here, such as xylem and pith tissues, could also undergo changes in gene expression that affect phenotypes related to cold tolerance. Furthermore, *MdKIN1*, *MdCOR47*, *MdSOC1*, and *MdSAG21*, which are related to cold tolerance via the CBF-dependent and CBF-independent pathways in apple^70^, were identified in several of the modules in response to chilling stress (Table S2); this finding provides evidence to support future cold tolerance research in apple.

## Materials and methods

### Materials and sample preparation

One-year-old branches from the grafted apple cultivars “Golden Delicious” (“G”) and “Jinhong” (“H”) in their ecodormancy stage in the field were sampled in February 2016. The branches of “G” were harvested from Xiongyue (40° 12′ N, 122° 07′ E), Liaoning Province, China, which is the northernmost geographical latitude of “G” cultivation. The highest temperature in January 2016 in this area was 4 °C, the lowest temperature was −22 °C, and the average temperature was −9.53 °C. The branches of “H” were sampled from Gongzhuling (43° 11′ N, 124° 02′ E), Jilin Province, China, where the highest temperature in January 2016 was 2 °C, the lowest temperature was −28 °C, and the average temperature was −15.16 °C. The northernmost latitude and longitude of “H” cultivation were 47°21′ N, 123°55′ E. The northernmost limit of “H” cultivation is 777 km north of the cultivation region of “G.” One-year-old branch samples were placed in a chamber and cold-stratified at 4 to 5 °C for 7 days. This treatment fully acclimated the samples to temperatures above the freezing point in order to study the gene expression characteristics following chilling and freezing stress treatments.

### Chilling and freezing stress treatments

Low-temperature stress was simulated by using a liquid-cooling programmable constant-temperature circulator (CKDC-4510: Fan Dilang Ltd, China), which can control the temperature with a 0 to 0.5 °C/min cooling speed and temperature gradations of ±0.1 to ±0.3 °C within a range of −45 to 100 °C. Branches of 20 cm length and uniform thickness were cut from the bases of the 1-year-old sampled branches and placed in closed stainless-steel boxes within the circulator. The electrolyte leakage rate of the apple branches in three biological replicates was measured by the electrical conductivity method to assess damage to branch cells during the post-thaw period^21,71^. The samples were maintained successively for 24 h at 4, −4, −9, −14, −19, −24, and −29 °C: three samples were maintained at 4 °C/24 h; three samples at 4 °C/24 h and then −4 °C/24 h; three samples at 4 °C/24 h, −4 °C/24 h and then −9 °C/24 h; three samples at 4 °C/24 h, −4 °C/24 h, −9 °C/24 h and then −14 °C/24 h; three samples at 4 °C/24 h, −4 °C/24 h, −9 °C/24 h, −14 °C/24 h and then −19 °C/24 h; three samples at 4 °C/24 h, −4 °C/24 h, −9 °C/24 h, −14 °C/24 h, −19 °C/24 h and then −24 °C/24 h; three samples at 4 °C/24 h, −4 °C/24 h, −9 °C/24 h, −14 °C/24 h, −19 °C/24 h, –24 °C/24 h and then −29 °C/24 h. The initial sample temperature of 4 °C was lowered at a rate of 5 °C/h until the desired temperatures noted previously were reached. The post-thaw branches were cut into small sections of 5 mm, and 3.0 g sections were weighed and put into trigonometric bottles with 30 ml ultrapure water. Then, each bottle was sealed with sealing film and placed onto a shaking table at 22 °C and 150 r/min for 20 h. The initial electrical conductivity was measured with an electric conductivity meter (DDBJ-350: Shanghai Precision Scientific Instrument Ltd, China). Subsequently, the samples were placed into a water bath and boiled for 20 min to kill the tissues; then, after 2 h of cooling to room temperature, the maximal electrical conductivity of the boiled branches was measured at room temperature. We obtained the electrical conductivity by calculating the ratio of the initial conductivity to the maximum conductivity. A cross-section of each stem was photographed using a stereomicroscope (SZ621: Olympus Ltd, Japan) and an imaging system (SMARTV950D, Charge Coupled Device: Youyuan Technology Development Ltd, China).

### RNA preparation

Two branch samples (two biological replicates) were flash-frozen in liquid nitrogen after being held successively at 4, −4, −9, −14, −19, −24, and −29 °C for 24 h, which were similar as the sample treatment for the electrolyte leakage rate measurement mentioned above. Then, the bark of the branches was removed and placed directly in liquid nitrogen. The scraped bark tissues included the bark epidermis, phloem, and cambium. Total RNA of the samples was extracted with an RNA Prep and Pure Plant Kit (DP441) suitable for polysaccharide- and polyphenolic-rich samples (Tiangen Biotech Co., Ltd, China). High-quality RNA samples were sequenced with the Illumina HiSeqX10 platform at NextOmics Co., Ltd. (Wuhan, China).

### Library preparation and RNA-sequencing transcriptome

We used 3 μg of RNA per sample as input material to construct the 28 sequencing libraries using the Illumina Gene Expression Sample Prep Kit. Sheared mRNA fragments were used as a template, and first-strand cDNA was synthesized with six random base hexamers. Second-strand cDNA was synthesized using dNTPs, RNase H, DNA polymerase I, and PCR buffer. mRNA was purified using the QiaQuick PCR kit, and end repair, poly (A) addition, and Illumina-indexed adaptor ligation were performed following the Illumina protocol. The libraries were sequenced on an Illumina HiSeqX10 platform, and 150 bp paired-end reads were generated. The amount of raw sequencing data per sample was >6 Gb.

### Quality control of RNA-sequencing transcriptome data

The NGSQC Toolkit (v2.3.3) (http://www.nipgr.res.in/ngsqctoolkit.html) was used for filtering the raw data (raw reads), and FastQC (http://www.bioinformatics.babraham.ac.uk/projects/fastqc/) software was used to perform quality control for the clean data. The Q30 of the clean data was calculated to verify the base quality.

### Transcriptome assembly

Clean reads were compared to the apple genome^1^ using TopHat^72^. The appropriate parameters of TopHat were set according to the gene model of the reference genome^1^. The filtered sequences were aligned with the reference genome, and the number of mapped reads was calculated. The clean read data were deposited in the National Genomics Data Center (NGDC) under the BioProject accession number PRJCA002421 (https://bigd.big.ac.cn/databases).

### Gene expression quantification and principal component analysis (PCA)

HTSeq v0.5.4p3 was used to count the number of reads mapped to each gene or exon region^73^. The read counts for each gene were normalized by the FPKM^74^ (fragments per kilobase of transcript per million mapped reads) method, which is often used for estimating gene expression levels. To reveal the variations in the datasets between the chilling and freezing stress levels, a cutoff of FPKM ≥ 0.01 was selected to define potentially meaningful gene expression. PCA was performed and visualized for each expressed gene (FPKM ≥ 0.01), and DEGs were identified with R software^32,75^. The DEGs between each chilling and freezing treatment group were identified using edgeR software^76^, in which “*p* ≤ 0.05” and “FDR ≤ 0.001” (false-discovery rate)^77^ were set as the threshold values for the significance of gene expression differences. Then, we extracted the union set of the DEGs between each chilling and freezing treatment group to obtain the DEGs expressed under each freezing stress treatment. Moreover, we identified DEGs in “Golden Delicious” (G) and “Jinhong” (H) by comparing the gene expression under each of the five freezing stress temperatures (−9, −14, −19, −24, and −29 °C) to the gene expression under the first freezing stress temperature (−4 °C). We set “fold change ≥ 2” and “*p* ≤ 0.05” as the thresholds for defining the significance of gene expression differences.

### Gene network construction

A gene coexpression network was constructed using the WGCNA^27^ (v1.64-1, https://horvath.genetics.ucla.edu/html/CoexpressionNetwork/Rpackages/WGCNA/index.html) package in R (v3.6.1). The FPKMs of “G” and “H” DEGs were used to construct the coexpression network. The modules were identified using the “step-by-step network construction and module detection” method with the default settings except that the soft-thresholding power was 8, the minModuleSize was 30, and the cut height for module merging was 0.25^27^.

The intramodular connectivity (*K*_ME_) is defined for the genes inside a given module and calculated as *K*_ME_ (*i*) = cor (xi, ME), where xi is the gene expression profile of gene *i* and ME is the module eigengene^27^. GS is defined as the gene significance and was measured with a function GS that assigns a number to each gene, “GS”^27^. We correlated the module eigengenes with LT, accumulated low temperature (Ac_LT) (the product of low temperature and its maintenance time), and the electrolyte leakage rate (ELR). Then, the correlation coefficients of GSs with LT, Ac_LT, and ELR were obtained and represented using the following abbreviations: GS_LT, GS_Ac_LT, and GS_ELR. We plotted scatter diagrams of the module eigengenes against LT, Ac_LT, and ELR and of GS against *K*_ME_ using the WGCNA package^27^.

### Hub gene identification and functional annotation

A gene union set in each module^27,78^ was identified from the intersections of the top 20% of genes by connectivity and GS_LT, connectivity and GS_ELR, or GS_LT and GS_ELR, and these genes were considered hub genes^27^. Pfam and transcription factor annotations were searched for the hub genes using Pfam software^52^ and PlantTFDB v5.0^79^. Gene ontology (GO) annotations of the hub genes were obtained utilizing TBtools^80^. Gene expression heatmaps and profile analysis were performed using OmicShare tools, a free online platform for data analysis (https://www.omicshare.com/tools/).

### Homologous gene alignment and qRT-PCR analysis

Gene sets related to the CBF^7,13,16^, ABA^57^, BR^64^, and ETH^66,67^ pathways defined recently in Arabidopsis were blasted (*E*-value ≤ e^−10^) against the DEGs and hub genes of apple using TBtools software^80^. Six hub genes with high *K*_ME_ from module 7 were selected for expression pattern validation. Gene-specific primers of the six hub genes were designed using Primer-BLAST (https://www.ncbi.nlm.nih.gov/tools/primer-blast/index.cgi). qRT-PCRs were completed in 25 µl reaction solutions consisting of 12.5 µl SYBR (Takara), 0.5 µl each specific primer, 2 µl cDNA diluted 20-fold and 9.5 µl ddH2O. qRT-PCR amplification of the 28 treatment samples with three technical replicates was performed with the following procedure: 95 °C for 30 s and 39 cycles of 95 °C for 5 s, 50 °C for 30 s and 72 °C for 30 s. *Mdtubulin*, an apple housekeeping gene, was used as an internal reference^81^. The expression ratio was calculated by the 2^−△△Ct^ formula, △△Ct = (Ct_target gene _− Ct_internal reference gene_) _freezing treatment_ − (Ct_target gene_ − Ct_internal reference gene_) _chilling treatment_, as previously described^82^.

## Supplementary information

Table S1 Summary of sequencing data quality and statistics of the transcriptome assembly

Table S2 The coexpression module, eigengene-based intramodular connectivity, and gene significance analysis

Table S3_7 Connectivity, gene significance (GS) and annotations of hub genes in module 7

Table S4_1 GO terms of hub genes responding to the chilling stress in module 1Table S4_1 GO terms of hub genes responding to the chilling stress in module 1

Table S5 Annotation analysis of apple’s hub genes and their homologous Arabidopsis’s genes related to CBF pathway in this paper

Table S6 Alignment results of apple’s hub genes with Arabidopsis Thaliana genome

Table S7 Gene ontology enrichment analysis of differentially expressed genes identified from freezing stresses

Table S8 Differentially expressed genes identified from freezing stresses and their homologous gene annotations in Arabidopsis

Table S9 qRT-PCR primer sequences and conserved protein domains of hub genes in module 7 and their homologous gene annotations in Arabidopsis Thaliana

Table S10 The alignment and annotation analysis of apple genome and DEGs in this study with CBF pathway-related genes in Arabidopsis

Table S11 The alignment and annotation analysis of apple genome and DEGs in this study with ABA pathway-related genes in Arabidopsis

Table S12 The alignment and annotation analysis of apple genome and DEGs in this study with BR and ETH pathway-related genes in Arabidopsis

Supplementary Figures
