# Supplementary material for: Transcriptional regulation of bark freezing tolerance in apple (Malus domestica Borkh.)
Source: Hortic Res. 2020 Dec 1;7:205. doi: 10.1038/s41438-020-00432-8 (PMC7705664; doi:10.1038/s41438-020-00432-8)
Supplement: Supplementary file 1 — Table S1 Summary of sequencing data quality and statistics of the transcriptome assembly [file 41438_2020_432_MOESM1_ESM.docx]

**Table S1 Summary of sequencing data quality and statistics of the transcriptome assembly**

| **Sample** | **Clean reads number** | **Clean bases number** | **Clean Q30 bases rate(%)** | **Mapped reads** | **Mapping rate(%)** |
| --- | --- | --- | --- | --- | --- |
| GC.1_1  GC.1_2 | 55,849,472  52,603,910 | 8.02G  7.66G | 96.67%  96.54% | 45,044,055  42,693,398 | 80.65%  81.16% |
| GF.1_1 | 56,775,984 | 8.25G | 96.59% | 46,183,787 | 81.34% |
| GF.1_2 | 61,693,782 | 8.95G | 96.59% | 49,945,773 | 80.96% |
| GF.2_1 | 55,429,762 | 8.06G | 96.67% | 45,056,640 | 81.29% |
| GF.2_2 | 60,782,136 | 8.85G | 96.46% | 50,716,952 | 83.44% |
| GF.3_1 | 57,635,522 | 8.39G | 96.35% | 46,014,827 | 79.84% |
| GF.3_2 | 56,726,658 | 8.21G | 96.46% | 45,986,308 | 81.07% |
| GF.4_1 | 60,401,312 | 8.78G | 96.09% | 48,368,755 | 80.08% |
| GF.4_2 | 58,431,204 | 8.52G | 95.80% | 46,357,730 | 79.34% |
| GF.5_1 | 58,138,636 | 8.52G | 95.98% | 46,196,881 | 79.46% |
| GF.5_2 | 53,549,132 | 7.87G | 95.64% | 42,139,436 | 78.69% |
| GF.6_1 | 60,692,108 | 8.77G | 96.31% | 48,217,333 | 79.45% |
| GF.6_2 | 53,699,600 | 7.83G | 96.02% | 42,339,771 | 78.85% |
| HC.1_1 | 58,820,352 | 8.51G | 95.84% | 43,506,950 | 73.97% |
| HC.1_2 | 55,412,616 | 8.06G | 95.72% | 40,895,535 | 73.80% |
| HF.1_1 | 59,493,180 | 8.58G | 96.08% | 45,114,657 | 75.83% |
| HF.1_2 | 58,022,786 | 8.43G | 95.86% | 43,936,555 | 75.72% |
| HF.2_1 | 50,0832,54 | 7.33G | 95.91% | 38,638,704 | 77.15% |
| HF.2_2 | 53,1315,56 | 7.76G | 95.77% | 40,026,805 | 75.34% |
| HF.3_1 | 54,9947,38 | 8.04G | 95.98% | 41,750,935 | 75.92% |
| HF.3_2 | 52,659,370 | 7.66G | 95.67% | 39,900,063 | 75.77% |
| HF.4_1 | 42,910,596 | 6.28G | 95.28% | 32,465,148 | 75.66% |
| HF.4_2 | 43,982,402 | 6.33G | 95.65% | 33,075,255 | 75.20% |
| HF.5_1 | 60,419,578 | 8.78G | 96.52% | 46,667,567 | 77.24% |
| HF.5_2 | 51,245,094 | 7.5G | 95.46% | 38,668,477 | 75.46% |
| HF.6_1 | 48,231,166 | 7.05G | 95.51% | 36,421,287 | 75.51% |
| HF.6_2 | 51,191,780 | 7.47G | 95.31% | 38,429,701 | 75.07% |

Notes: The low-quality reads were discarded, and the clean reads were obtained by using NGSQC Toolkit and FastQC software. A total of about 693 million clean pair-end reads with lengths of 150 bp were generated. Clean reads were mapped to the *Malus domestica* genome using TopHat V 2.1.1 (http://ccb.jhu.edu/software/tophat/index.shtml). 80.40% and 75.55% of the total clean reads from ‘Golden Delicious’ and ‘Jinhong’ apples were uniquely mapped to the *Malus domestica* genome, respectively.
