# Supplementary material for: Transcriptional regulation of bark freezing tolerance in apple (Malus domestica Borkh.)
Source: Hortic Res. 2020 Dec 1;7:205. doi: 10.1038/s41438-020-00432-8 (PMC7705664; doi:10.1038/s41438-020-00432-8)
Supplement: Supplementary file 13 — Supplementary Figures [file 41438_2020_432_MOESM13_ESM.docx]

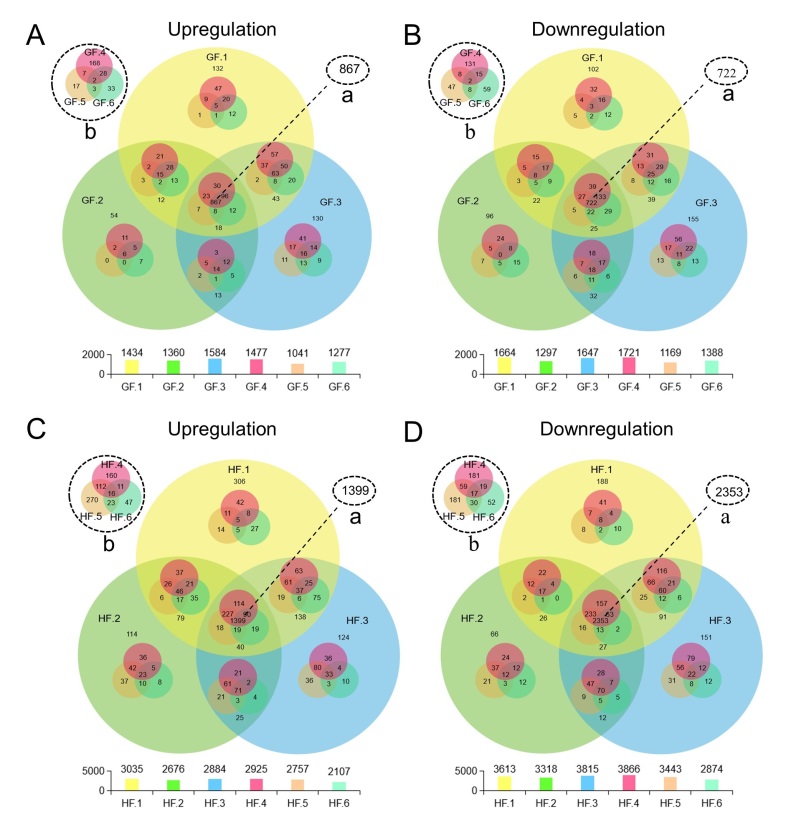


**Figure S1. Differentially expressed genes (DEGs) in response to freezing stress in one-year-old branches of ‘Golden Delicious’ (G) and ‘Jihong’ (H) apple trees.** The numbers in each large circle represent the total number of upregulated or downregulated DEGs in each treatment. The overlapping parts of the circles represent mutual DEGs between different treatments. **A-B** Up- and downregulated DEG numbers for ‘G’. GF.1, GF.2, GF.3, GF.4, GF.5, and GF.6 represent ‘G’ freezing stress treatments at -4 °C, -9 °C, -14 °C, -19 °C, -24 °C, and -29 °C; **C-D** Up- and downregulated DEG numbers for ‘H’. HF.1, HF.2, HF.3, HF.4, HF.5, and HF.6 represent ‘H’ freezing stress treatments at -4 °C, -9 °C, -14 °C, -19 °C, -24 °C, and -29 °C. ‘a’ shows the DEGs in response to the freezing stress at -4 °C, -9 °C, -14 °C, -19 °C, -24 °C, and -29 °C; ‘b’ shows the DEGs in response to freezing stress at -19 °C, -24 °C, and -29 °C.


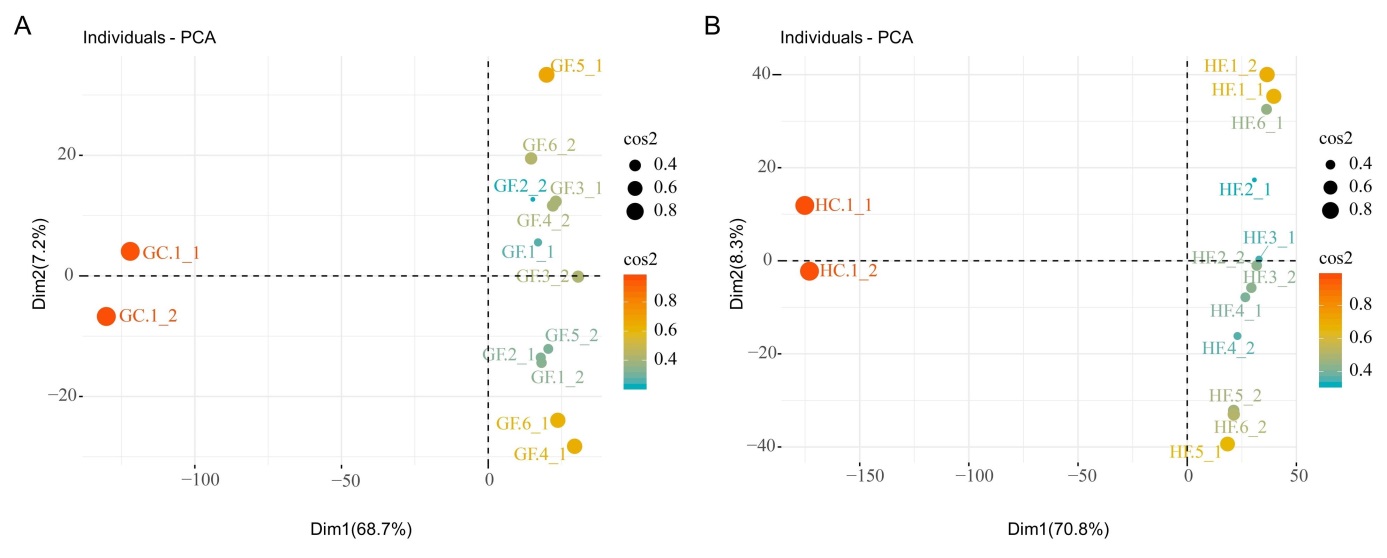


**Figure S2. Principal component analysis (PCA) of differentially expressed genes (DEGs) in ‘Golden Delicious’ (G) and ‘Jinhong’ (H) apple, respectively. A** PCA results for 4,173 DEGs in ‘G’. **B** PCA results for 7,734 DEGs in ‘H’. GC and HC represent the chilling treatments applied to ‘G’ and ‘H’; GF and HF represent the freezing treatments applied to ‘G’ and ‘H’.


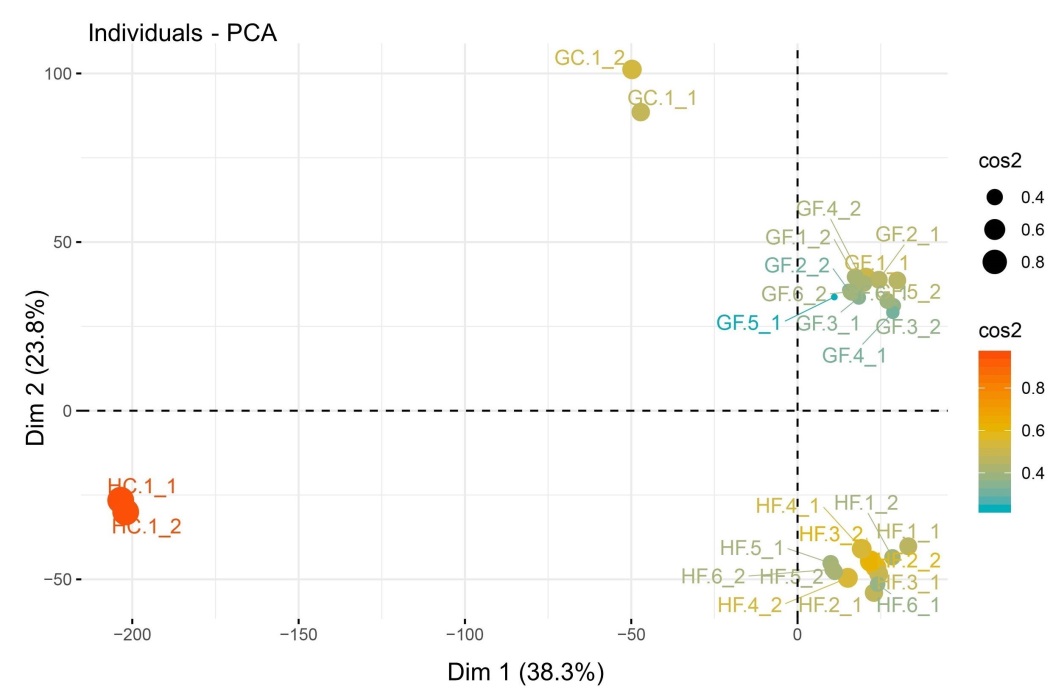


**Figure S3 Principal component analysis (PCA) of the differentially expressed genes (DEGs) in ‘Golden Delicious’ (G) and ‘Jinhong’ (H) apple, including 4,173 DEGs in ‘G’ and 7,734 DEGs in ‘H’.** GC and HC represent the chilling treatments applied to ‘G’ and ‘H’; GF and HF represent the freezing treatments applied to ‘G’ and ‘H’.


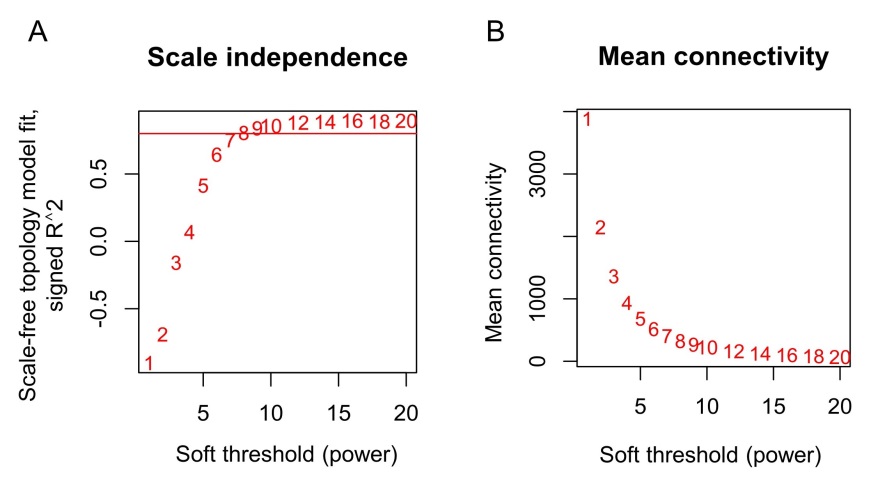


**Figure S4 Scale-free topology model fitting of gene coexpression modules of differentially expressed genes (DEGs) by using the weighted gene correlation network analysis (WGCNA) method. A** The scale-free fit index (y-axis) as a function of the soft threshold (power) (x-axis). **B** The mean connectivity (degree, y-axis) as a function of the soft threshold (power) (x-axis). Plot showing the scale-free topology *R*^2^ values as a function of the increasing soft thresholding power and the relationship between mean connectivity and the soft threshold.


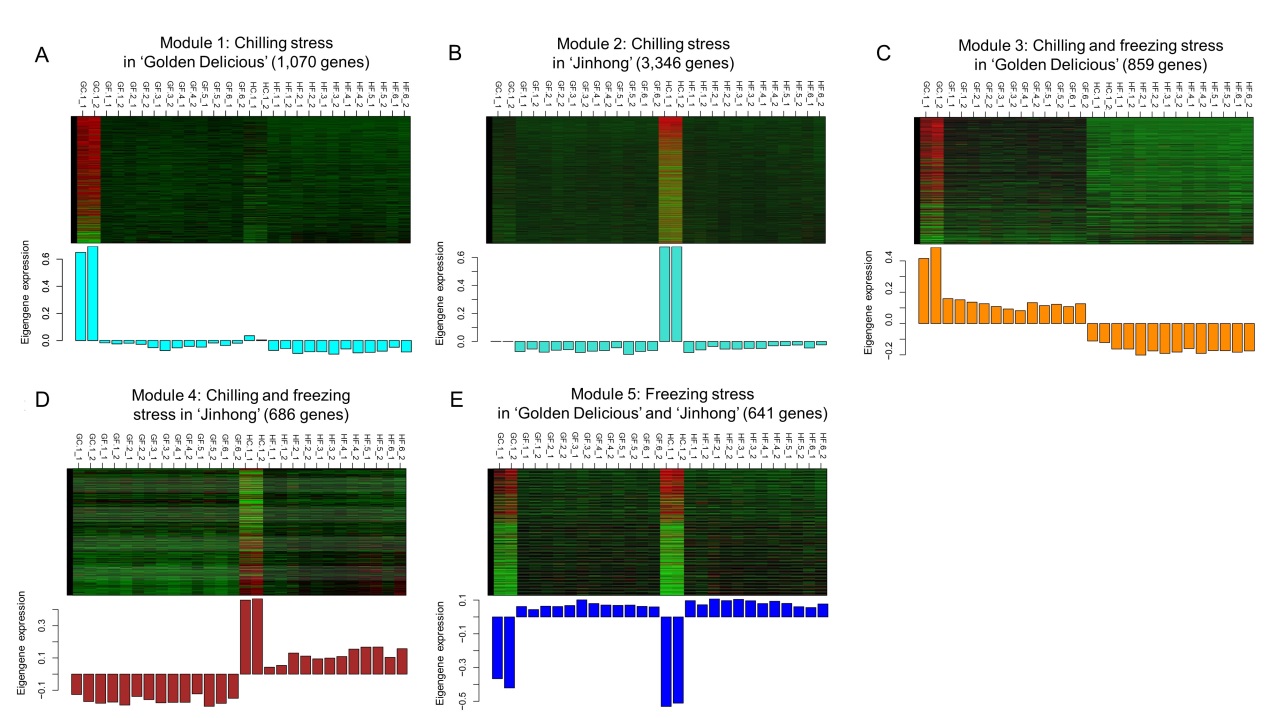


**Figure S5 Expression heatmaps of** **differentially expressed genes and expression profiles of eigengenes in modules 1, 2, 3, 4 and 5. A** Module 1, in response to chilling stress in ‘Golden Delicious’ (G). **B** Module 2, to chilling in ‘Jinhong’ (H). **C** Module 3, to chilling and freezing in ‘G’. **D** Module 4, to chilling and freezing in ‘H’. **E** Module 5, to freezing in ‘G’ and ‘H’. In the heatmap, rows represent the module genes, and columns indicate the stress treatment samples. Red indicates upregulated genes, and green indicates downregulated genes. GC and HC represent the chilling treatments applied to ‘G’ and ‘H’; GF and HF represent the freezing treatments applied to ‘G’ and ‘H’. The eigengene expression in WGCNA is defined as the first principal component of a given module, which can be representative of the gene expression profiles in a module. The bar graph of eigengene expression shows the eigengene value variance calculated from the singular value composition for each module.

**
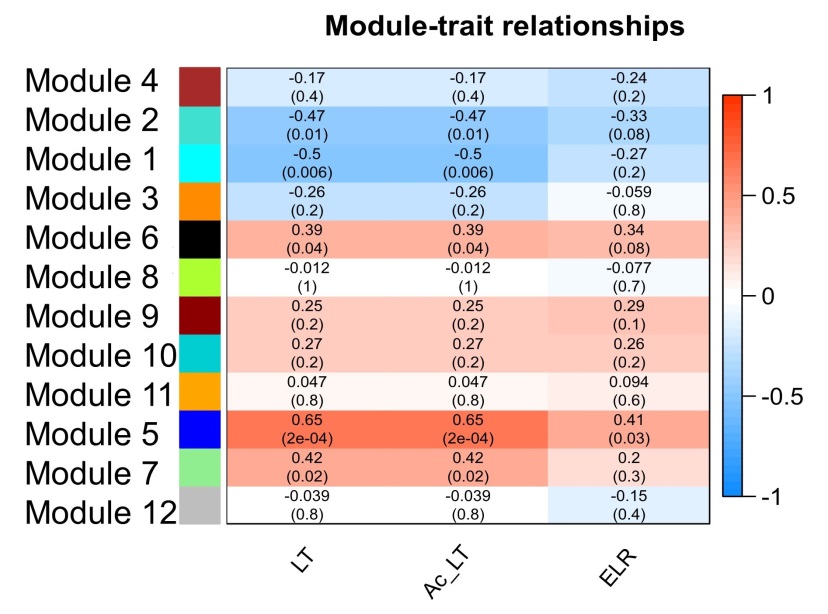
**

**Figure S6 Heatmap of the module-trait relationships.** Each row corresponds to a module eigengene, and each column corresponds to a trait. Each cell contains the correlation coefficient and the corresponding *p* value between the module and the trait. The figure is color-coded by correlation according to the color legend on the right. *LT*, low temperature; *Ac_LT*, accumulated low temperature; *ELR*, electrolytic leakage rate.


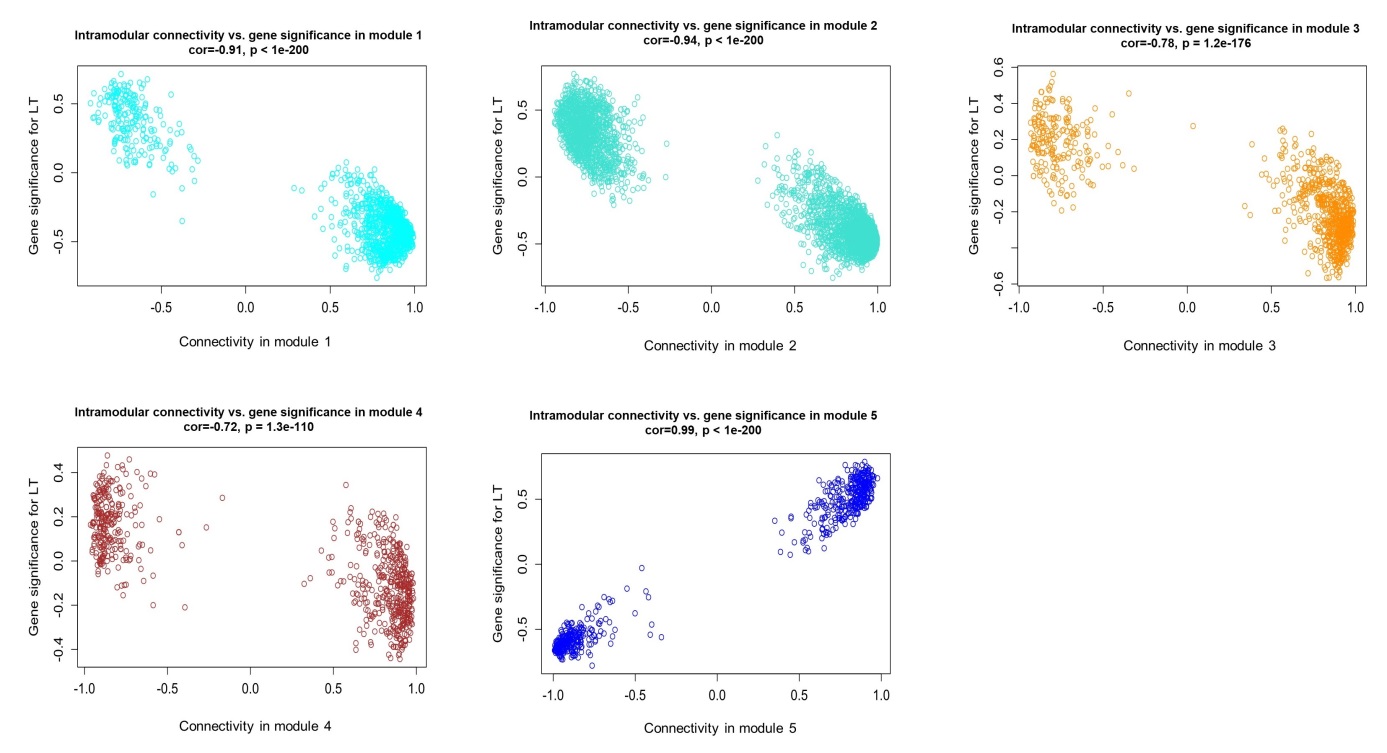


**Figure S7. The gene significance for low temperature (y-axis) vs. the intramodular connectivity (x-axis) plotted separately in modules 1, 2, 3, 4, and 5.** Gene significance (*GS*) for low temperature (*LT*) means a low temperature-based gene significance obtained by correlating the modules to *LT* values with the eigengene network methodology. We observed that the intramodular hub genes in module 5 showed a positive correlation between gene significance and intramodular connectivity. The opposite relationship was clear in modules 1, 2, 3, and 4. The intramodular connectivity (*K_ME_*) is the module eigengene-based network connectivity.


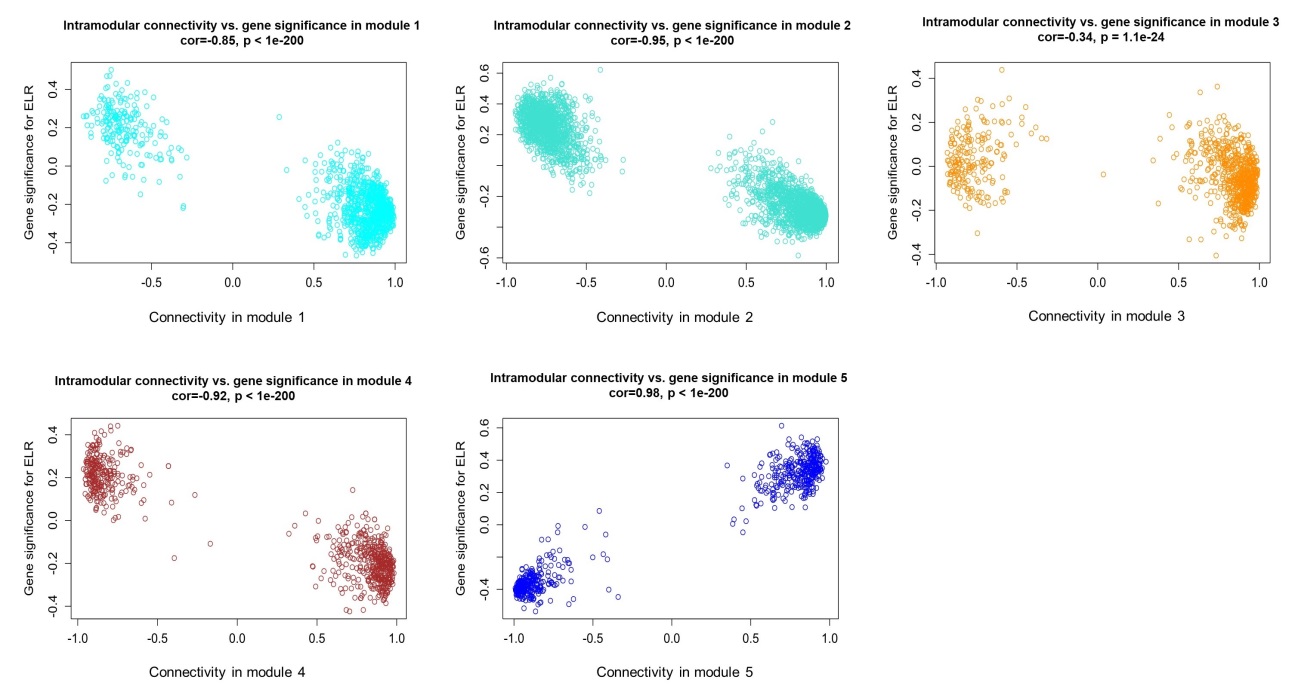


**Figure S8. The gene significance for electrolytic leakage rate (y-axis) vs. the intramodular connectivity (x-axis) plotted separately in modules 1, 2, 3, 4, and 5.** Gene significance (*GS*) for electrolytic leakage rate (*ELR*) means the electrolyte leakage rate-based gene significance obtained by correlating the modules to *ELR* values of the apple branches by using the eigengene network methodology. We observed that the intramodular hub genes in module 5 had positive correlations between gene significance and intramodular connectivity. The opposite relationship was observed in modules 1, 2, 3, and 4. The intramodular connectivity (*K_ME_*) is the module eigengene-based network connectivity.


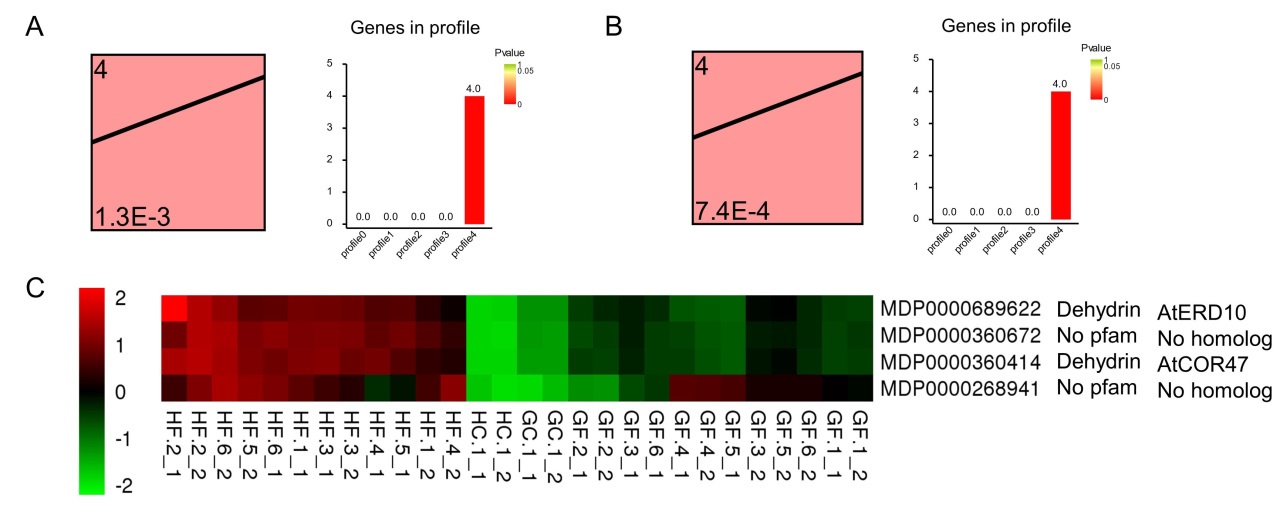


**Figure S9 The expression profiles and heatmaps of hub genes in response to water (GO:0009415) in module 7. A-B** The trends and expression profile statistics of the hub genes of ‘G’ and ‘H’. The trend diagram on the left presents the most significant gene expression trends, including the gene expression profile number on the top left and its *p* values on the bottom left. The bar graph on the right indicates the gene number assigned to one gene expression profile, and its color reveals the *p* values of the gene assignment significance. **C** The hub gene expression heatmaps for module 7. The columns represent 28 samples of ‘G’ and ‘H’ treated with chilling (C) and freezing (F) stress in this study. The rows show the hub genes and their types. Red in the heatmaps indicates upregulated expression, and green indicates downregulated expression.


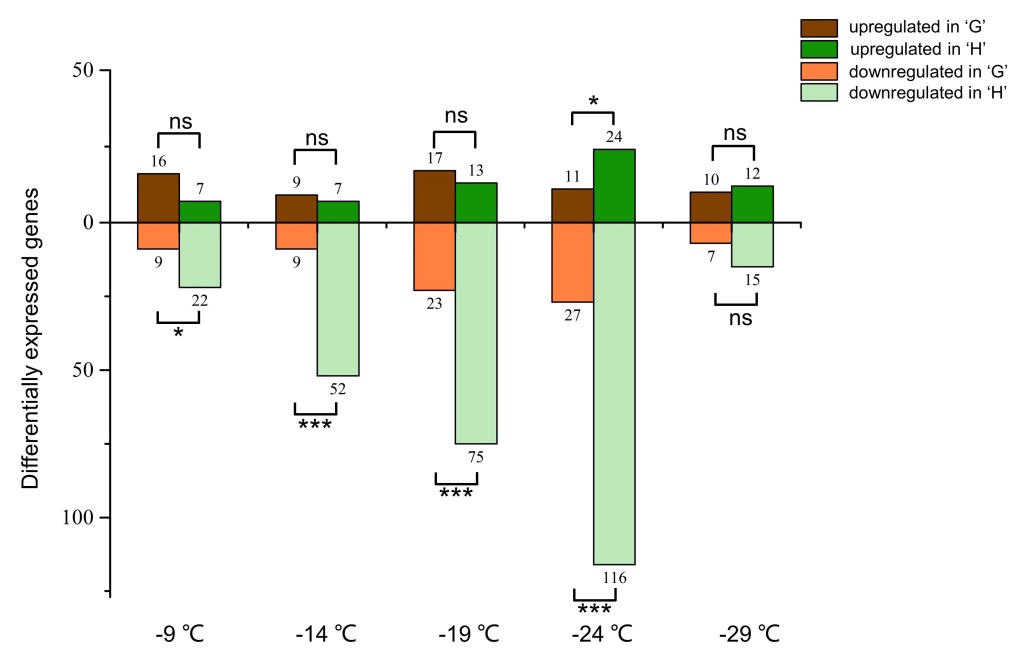


**Figure S10 Number of differentially expressed genes (DEGs) identified between the five colder freezing stress treatments (-9 °C, -14 °C, -19 °C, -24 °C, and -29 °C) and the first freezing stress treatment (-4 °C) in ‘Golden Delicious’ (G) and ‘Jinhong’ (H).** A fold change ≥ 2 was set as the threshold for defining the significance of the gene expression differences. ‘*’ and ‘***’ indicate significance at the 0.05 and 0.001 levels by the chi-square test of the difference in DEG numbers between ‘G’ and ‘H’ samples at the same temperature. ‘ns’, no significance according to the chi-square test.
